# Advanced nanomaterials for highly efficient CO_2_ photoreduction and photocatalytic hydrogen evolution

**DOI:** 10.1080/14686996.2022.2149036

**Published:** 2022-12-08

**Authors:** Rashmi Nautiyal, Deepika Tavar, Ulka Suryavanshi, Gurwinder Singh, Archana Singh, Ajayan Vinu, Gurudas P. Mane

**Affiliations:** a Department of Chemistry, Sunandan Divatia School of Science, SVKM’s NMIMS (Deemed-to-be) University, Mumbai, India; b Academy of Scientific & Innovative Research (AcSIR), Ghaziabad, India; c Center for Advanced Radiation Shielding and Geopolymeric Material, CSIR– Advanced Material and Processes Research Institute, Bhopal, India; d Rayat Shikshan Sanstha’s, Karmveer Bhaurao Patil College, Vashi, Navi Mumbai, India; e Global Innovative Centre for Advanced Nanomaterials, School of Engineering, College of Engineering, Science, and Environment, The University of Newcastle, Callaghan, NSW, Australia

**Keywords:** Photocatalysis, nanomaterials, CO_2_ reduction, water splitting, hydrogen generation, TiO_2_, non-TiO_2_ materials

## Abstract

At present, CO_2_ photoreduction to value-added chemicals/fuels and photocatalytic hydrogen generation by water splitting are the most promising reactions to fix two main issues simultaneously, rising CO_2_ levels and never-lasting energy demand. CO_2_, a major contributor to greenhouse gases (GHGs) with about 65% of the total emission, is known to cause adverse effects like global temperature change, ocean acidification, greenhouse effects, etc. The idea of CO_2_ capture and its conversion to hydrocarbons can control the further rise of CO_2_ levels and help in producing alternative fuels that have several further applications. On the other hand, hydrogen being a zero-emission fuel is considered as a clean and sustainable form of energy that holds great promise for various industrial applications. The current review focuses on the discussion of the recent progress made in designing efficient photocatalytic materials for CO_2_ photoreduction and hydrogen evolution reaction (HER). The scope of the current study is limited to the TiO_2_ and non-TiO_2_ based advanced nanomaterials (i.e. metal chalcogenides, MOFs, carbon nitrides, single-atom catalysts, and low-dimensional nanomaterials). In detail, the influence of important factors that affect the performance of these photocatalysts towards CO_2_ photoreduction and HER is reviewed. Special attention is also given in this review to provide a brief account of CO_2_ adsorption modes on the catalyst surface and its subsequent reduction pathways/product selectivity. Finally, the review is concluded with additional outlooks regarding upcoming research on promising nanomaterials and reactor design strategies for increasing the efficiency of the photoreactions.

## GRAPHICAL ABSTRACT

**Figure:**
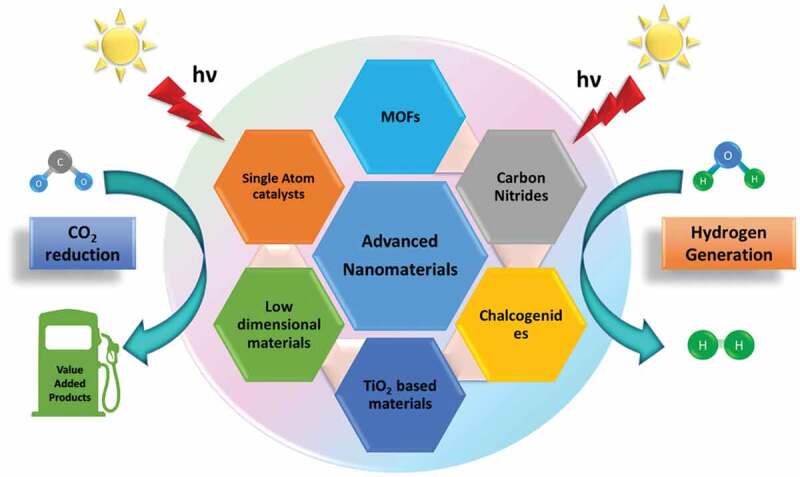

## 1. Introduction

In the last few decades, air pollution tragedies can be well credited to the continuous exploration and combustion of fossil fuels (including coal, oil, and natural gas). These fossil fuels still lead the energy market for different purposes, including electricity/heat generation, transportation, industrial sector, and to some extent for, residential use. Their utilization releases large amounts of CO_2_, which is regarded as one of the primary greenhouse gases (GHGs) and its emission through all these anthropogenic activities makes it a key air pollutant [1,2]. The fuel combustion procedures, natural gas processing, energy generation units (EGUs), and other industrial practices are considered as large stationary sources of CO_2_ emission (>0.1 Metric Tons/year). The rising levels of CO_2_ not only contribute to pollution but also cause adverse effects like an upsurge in global warming, lowering of the seawater pH, and climate change [3,4]. In the last 100 years, there has been a global rise in CO_2_ concentration (80 ppm) and a corresponding increase in global temperature by 1.5° C, which is an alarming situation. As stated above, the emitted CO_2_ not only leads to air pollution but also decreases the alkalinity of ocean water, which severely affects calcifying ocean organisms such as corals, coccolithophores (single-celled algae), and crustaceans, contributing to water pollution too. The synthetic report issued by the IPCC, i.e. the Intergovernmental Panel of Climatic Change, in the year 2014 highlights the risk associated with anthropogenic Greenhouse gases (GHG) activities [5] mostly due to stationary sources like fossil fuel-based electric power plants, independent power producers, industrial manufacturers of cement, limestone, etc., and mobile sources of CO_2_ emissions that include different modes of transportation [6]. It becomes significantly important to find immediate remedies to control and minimize the CO_2_ concentration in the atmosphere [7]. One of the feasible methods to address the CO_2_ problem is to capture it before being released into the atmosphere, as such technologies form an integral part of several industries [8–13]. Depending upon the type and contents of the flue gas stream in the industries, CO_2_ capture could be generally classified into three types: post-combustion CO_2_ capture, pre-combustion capture, and oxyfuel combustion. The technical and economic aspects of these methods are well documented by Kanniche et al. in a review article [14]. Another attractive prospect is to use CO_2_ as a valuable feedstock for various catalysis reactions, resulting in the formation of useful products.

### 1.1. Fundamentals of CO_2_ conversion

**Figure 1.**
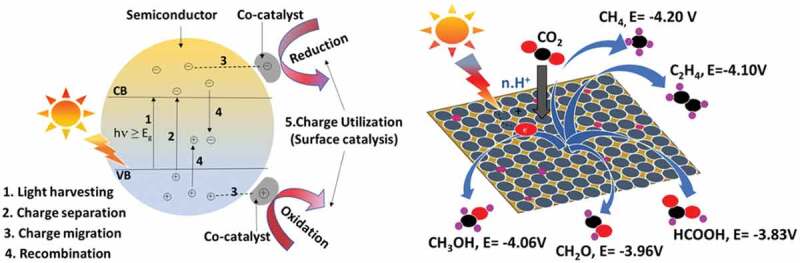
Schematic representation of and stepwise mechanism in photocatalytic reactions (left) and CO_2_ photoreduction to chemical fuels on the surface of metal-doped photocatalyst (right). *Source*: [18,19].

One of the most important methods is the photocatalytic reduction of CO_2_ to produce solar fuels (methane or methanol) and other useful chemicals [15]. These fuels store energy in the form of C-H bonds when combusted or used in fuel cells and release back CO_2_ to complete the carbon cycle. In this reaction, water or water vapor is used as a proton source which splits under solar light irradiation and supplies hydrogen for CO_2_ hydrogenation. Typically, when a semiconductor catalyst absorbs photons with energy greater than its band gap, electrons are excited from the valence band (VB) to the conduction band (CB), leaving behind quasi-particles called holes. These electrons and holes then migrate to the surface, where they get utilized by the reactant molecules to complete the reduction and oxidation reactions, respectively [16,17] (Figure 1). In the case of CO_2_ photoreduction, the reactant molecule is CO_2_ (adsorbed on the surface of the semiconductor), and photogenerated electrons need to be transferred from CB to the antibonding orbital of adsorbed CO_2_, while holes oxidize water and produce O_2_ or H_2_O_2_. The oxidation half-reaction is difficult to occur and is taken care of by additional hole scavengers; therefore, the focus reaction for CO_2_ conversion is mainly the reduction half. However, CO_2_ photoreduction is also an uphill reaction (Gibbs free energy, ΔG^0^ >0), which means that it requires substantial energy to make it happen (in this case, it is supplied by photons). In addition, several factors can affect the rate of CO_2_ photoreduction and product selectivity, which will be discussed in a separate section of this review.

### 1.2. Hydrogen as an alternate fuel

While looking at the possibilities of clean fuels that possess the potential to replace fossil fuels, hydrogen is regarded as one of the primary contenders, both by researchers and governments all over the world. One of the main advantages of hydrogen as a fuel is its comparatively higher heat value (120–142 MJ/Kg) as compared with conventional fossil fuels (40–50 MJ/Kg) and most other fuels [20,21]. More significantly, hydrogen is a very clean and eco-friendly fuel that emits zero greenhouse gases during combustion.

**Figure 2.**
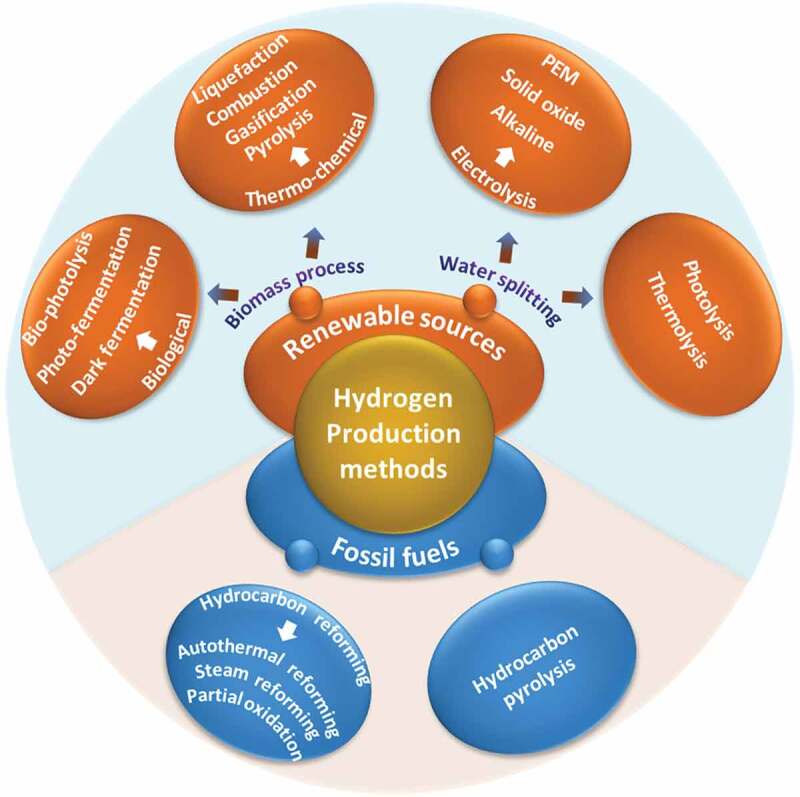
Different routes for Hydrogen production using conventional and renewable energy sources. *Source*: [24].

Hydrogen gas also has a large-scale demand in industries due to its exciting properties like non-polluting, non-decomposing, nontoxic, and non-corrosive nature [22] for various chemicals and petrochemical applications like refineries (25%), ammonia production (55%), methanol production (10%), and other miscellaneous purposes (10%) [23]. The gas can be produced using coal, oil, and natural gas feedstocks. At present, nearly 6% of global natural gas and 2% of global coal are utilized for hydrogen generation. However, the use of natural gas for H_2_ generation leads to a debate on economic and technical issues. Fuel costs accounting for 45–75% of the total hydrogen production make the process costlier [24]. The other applications worldwide account only for 10% of global hydrogen production. However, the properties like low ignition rate, fast flame speed, broad range of flammability, and low energy density make it difficult for consumers to store and safely transport hydrogen fuel. These concerns related to storage and handling are now getting addressed with a large positive momentum for transforming hydrogen into hydrogen-based fuels and feedstock, which are easy to stock, transport and utilize. On the other hand, photocatalytic splitting of water to produce hydrogen by using a suitable semiconductor catalyst brings several advantages on board. Compared with other methods (Figure 2), photocatalytic HER is more facile, inexpensive, and generates ‘green’ hydrogen, i.e. solar light is used to split water into hydrogen and oxygen without any negative impact on the environment [25].

When narrow band gap semiconductors (SCs) are irradiated with visible light, electrons are excited from VB to CB. Photocatalytic H_2_ generation and CO_2_ photoreduction reactions make use of these CB electrons for reducing H_2_O and CO_2_ reactant molecules, respectively. Conceptually, both processes share similar initial steps: 1) light harvesting by the SC catalyst, 2) electron–hole (e-/h+) pair formation, 3) separation of these (e-/h+) pairs and migration to the surface of the SC catalyst. The major difference lies in the last step of surface reaction where these migrated charge carriers react with adsorbed reactants (CO_2_ and H_2_O) [18]. Photocatalytic H_2_ generation is a thermodynamically and kinetically feasible reaction wherein the required potential for H+ generation from water is −0.42 eV (vs SHE, pH 7). However, CO_2_ photoreduction is a complex reaction involving energy-intensive steps such as CO_2_ adsorption on the catalyst surface, breaking of strong C=O bond, and formation of new bonds (C-H, C-C) during these surface reactions. For converting CO_2_ into high carbon content fuel (e.g. CH_4_), generally water (liquid or vapor) is used as a proton source. Since both reactions need similar reduction potentials, there is continuous competition for extracting electrons from CB for water splitting and CO_2_ reduction, and kinetically, H+ reduction is more feasible. If sufficient protons are provided, proton-assisted multi-electron transfer is more feasible thermodynamically (e.g. CO_2_ to CH_4_ conversion, at −0.24 eV, SHE, pH-7), when compared with the formation of CO_2_^−ׄ•^ by one-electron reduction (−1.9 eV vs SHE, pH-7). Another difference is in the product formation, and water splitting reaction produces defined products; H_2_ and O_2_ but CO_2_ photoreduction can lead to various products in gas/liquid state, radical intermediates, and the actual mechanism has not been fully understood. More details are described in the respective sections below.

**Figure 3.**
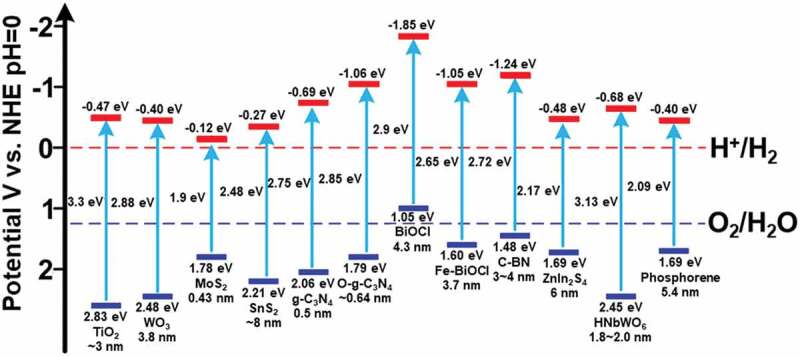
Bandgap energies and band edge positions of several photocatalysts for water splitting. (Reprinted with permission from Ref [16]. Copyrights 2018 American Chemical Society).

The coupling of the co-catalyst with the semiconductor has been observed to enhance the performance of photo reactions due to CO_2_ activation and charge separation [26]. These cocatalysts could be noble metals (e.g. Pt and Au), transition metals (e.g. Cu and Ni) or metal oxides (e.g. NiO and RuO_2_) in nanoform. The bandgap energies and corresponding band edge positions of several photocatalysts for water splitting are shown in (Figure 3).

For the photocatalytic reaction to occur, the VB edge of the catalyst should be more positive than the oxidation potential (H_2_O to O_2_), whereas the CB edge should be more negative than the reduction potential (H+ to H_2_) [27]. The materials like O-g-C_3_N_4_, SnS_2_, C-BN, ZnIn_2_S_4_ possess a suitable combination of band gap energy and redox potentials (Figure 2) to carry out a water splitting reaction. However, to achieve a high rate of green hydrogen production, hurdles like fast recombination of photogenerated charge carriers and poor visible light absorption need to be resolved [28]. Hisatomi et al. discussed other important problems in large-scale solar hydrogen production and reactor design in their recent review [27].

The current review focuses on the latest developments in catalyst materials with respect to their photocatalytic activity for CO_2_ reduction and H_2_ generation. The various parameters that control the process of CO_2_ photoreduction and photocatalytic H_2_ generation are discussed in detail. The H_2_ production is possible through different routes (Figure 3), with each one having its pros and cons, which are listed in Table 1. Enormous progress has been made on photocatalytic CO_2_ reduction/H_2_ generation, and the review discusses the performance of various nanomaterials for these reactions. The review concludes by providing a summary of the research status in the field and directions for future research.

**Table 1.** Advantages, disadvantages, and efficiency of various hydrogen production routes.

Hydrogen production method	Advantages	Disadvantages	Ref.
Steam reforming	The overall reaction converts CH_4_ to CO_2_ and H_2,_ increasing hydrogen efficiency	Deactivation of the catalyst due to coking or sintering	[29]
Partial oxidation	Good response time	Undergoes slow kinetics and has coking properties.	[30]
Autothermal reforming	Reduction in the coke formation rate	High energy consumption	[31]
Biophotolysis	Solar energy is converted into chemical energy using renewable and sustainable sources	Non-utilization of waste and low hydrogen production	[32]
Dark fermentation	Low energy demand, compatible with a wide range of biomass as feedstocks	Low hydrogen gas production due to the formation of various final products.	[33]
Photo fermentation	Low environmental pollution and cost-effective	Inhibitory compounds present in biological substrates affect the action of PNS bacteria reducing the hydrogen efficiency	[34]
Steam gasification	High gasification rate and low ash production	Purification and separation of gaseous products are difficult	[35]
Pyrolysis	Cost-effective due to cheap feedstocks	Low H_2_ partial pressure leads to weak hydrogen separation	[36]
Thermolysis	Sustainable process, O_2_is the only by-product	Temperature beyond 2500° C is required, leading to dissociation of only 30–35% water	[37]
Electrolysis	Less GHGs are emitted if renewable sources are used	Expensive technique with lower fuel efficiency	[38]

The following sections will describe in detail the CO_2_ capture, adsorption and conversion pathways and the role of different factors that affect the CO_2_ conversion/H_2_ generation processes.

## 2. CO_2_ photoreduction

The evolution of the Sabatier reaction (methanation) gave researchers another outlook of CO_2_ reduction to form methane and water in the presence of nickel catalyst; however, further modification with ruthenium and palladium proved to be the efficient catalysts in terms of activity and selectivity, respectively [39]. If the activity of the catalyst is optimized so that methanation becomes gas selective (CO_2_, N_2_, Ar – Mars’ abundant gases), the process can be used to produce water onboard for Mars exploration as recommended by NASA [40]. While the process seems efficient, it inherits some challenges that include:

- Large overpotential – Doping or introducing foreign material into the matrix is considered as the effective approach to overcome the large overpotential [41]
- Poor product selectivity – The competing reaction, i.e. the H_2_ evolution, is the major reason behind the poor selectivity of the product in CO_2_ reduction reaction (CO_2_RR) [42]. To overcome this issue and enhance product selectivity, factors like light absorption, band structures, charge separation efficiency, adsorption and activation of starting product, and surface catalytic reactions followed by adsorption/desorption of the formed intermediates can be optimized [43].
- High recombination rates – The recombination rate is higher in CO_2_RR majorly due to the overlapping of VB and CB potentials, which can be controlled by adding a co-catalyst. The addition of a co-catalyst improves charge separation, suppresses back reactions and increases the stability of the main catalyst. Recently, Ran et al. comprehensively reviewed the performance of various co-catalysts towards CO_2_ reduction reaction [44]. Another technique through which the recombination rate can be controlled is by using an organic or inorganic hole scavenger which will act as an electron donor, thereby scavenging the no. of holes formed due to the competing H_2_ reaction [45].

Apart from the above-mentioned factors, other thermodynamic and kinetic challenges discussed in the next section need equal attention when developing a catalytic system for the CO_2_ reduction reaction.

### 2.1. Factors affecting CO_2_ photoreduction

Various thermodynamic and kinetic parameters hold significant importance for CO_2_ photoreduction. Some of these are discussed below:

#### 2.1.1. Bandgap position

**Figure 4.**
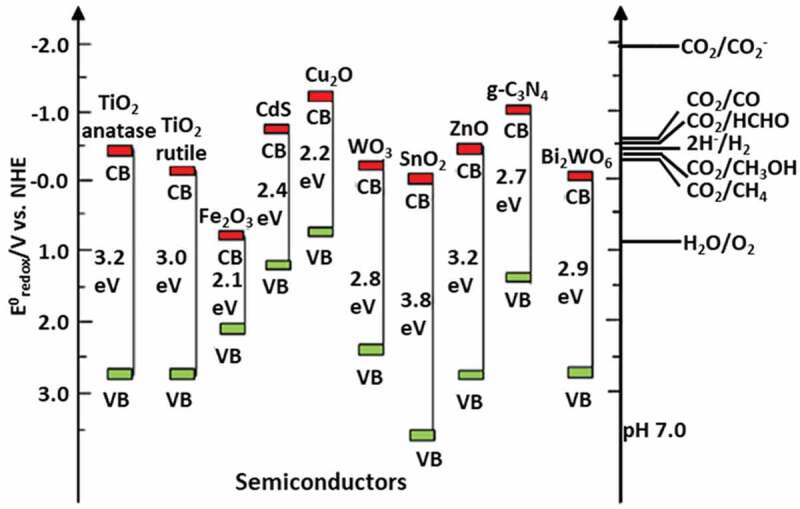
Redox potentials and bandgap of various photocatalysts with respect to different chemical species measured at pH 7. (Reprinted with permission from Ref [17]. Copyrights 2019 Elsevier).

Linear-shaped CO_2_ molecule is very stable with less electron affinity and zero dipole moment (C=O, ΔH_f_ ≈ −394.4 kJ mol^−1^). This restricts the electron transfer process; hence, CO_2_ reduction requires a significant energy input (ΔG > 0). One of the possible routes for CO_2_ conversion is the photocatalytic reduction of CO_2_ wherein bending its linear shape and photoelectron transfer to the antibonding orbital is realized. As discussed in the above section for photocatalytic H_2_ evolution, to achieve a high rate of CO_2_ photoreduction, the CB edge of the semiconductor photocatalyst must have negative potential with respect to the typical CO_2_ reduction potential (or H+/H_2,_ (Figure 4)) and VB should display more positive potential as compared to water oxidation potential (O_2_/H_2_O). A high reductive potential (−1.9 V vs. NHE) is required for CO_2_ to CO_2_- reduction, which is extremely unlikely. However, the required potentials are comparatively lower for two electron reduction (CO_2_/CO, −0.53 V) and proton-assisted multi-electron reductions.

For CO_2_ photoreduction, the similarity in the reduction potential (Figure 4) for a wide range of fuels raises a challenge in attaining product selectivity and allows exploring computational screening [18]. In the case of the narrow bandgap materials, they can absorb 43% visible light and 52% of infrared light of the total solar radiation but have to simultaneously possess appropriate band edge locations for continuous CO_2_ hydrogenation reaction [46]. To address this, researchers use sacrificial agents for matching the standard potentials. When the VB position is negative compared to the typical potential of H_2_O oxidation, the sacrificial agents facilitate CO_2_ photoreduction. The use of such electron donors not only ensures less recombination rates by scavenging the holes produced during the reaction but also increases the efficiency of CO_2_ photoreduction by speeding up the separation rates of electron–hole pairs [47]. Some commonly used sacrificial agents are tertiary amine [48], triethanolamine [49], methanol [50], and ethanol [51]. In order to further achieve better performance of photocatalysts, several strategies were implemented. One of the well-researched approaches is doping the parent photocatalytic materials with suitable foreign elements.

#### 2.1.2. Doping

Introducing foreign materials like metals, non-metals, or semiconductors to enhance electron transport is another technique for engineering bandgap. As discussed above, the redox potentials play an essential role for a reaction to occur. When the photocatalyst is doped with foreign materials, the bandgap can be tuned which induces a change in CB position and thereby facilitates light absorption and subsequent redox reactions [19,52]. The phenomenon of change in energy states and bandgap is explained by Moss–Burstein effect in detail [53]. For example, nitrogen-doped CeO_2_ showed properties like lower band gap energy, enhanced CO= adsorption capacity, and higher surface Ce^3+^ concentration with more oxygen vacancies. It showed an extended light spectral response and reduced charge recombination, leading to superior photocatalytic activity [54]. In a recent study, Sayed et al. synthesized multi-shell (Mn, C-co-doped) ZnO hollow spheres using the coordination polymer-assisted solvothermal method [55]. Mn-doped samples showed more CO_2_ adsorption capacity than C-ZnO due to the large quantity of surface oxygen species and CO_2_ activation by Mn ions. This multi-shell catalyst (2% Mn C-ZnO) showed a CO production rate of 0.83 µmol/g, two times higher compared to commercial ZnO. The authors credit this enhanced efficiency to increased light harvesting ability from a unique shell structure, the switchable valence state of Mn that promotes CO_2_ activation and the pathway for photogenerated electrons to reach the conduction band of ZnO. Reza et al. synthesized TiO_2_ catalysts with Ni, Bi doping and Ni-Bi co-doping by using a sol–gel approach. The co-doped sample showed 6.5 times more methane production than pure TiO_2_. A narrow band gap from the DRS study and decreased intensity in PL spectra of doped/co-doped samples can be related to increased efficiency [56]. Moradi et al. further confirmed the hypothesis by reporting a reduction in recombination when Pt and Bi were co-doped in the TiO_2_ matrix. The results obtained by PL studies indicate the superior ability of Pt for trapping photogenerated electrons [57].

**Figure 5.**
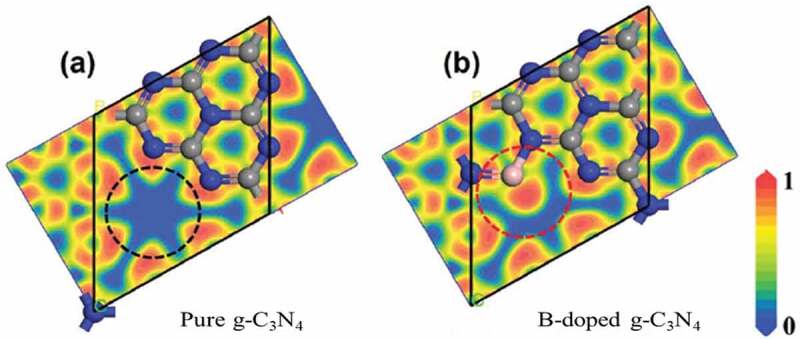
Electronic localization function of a) pure g-C_3_N_4_ and b) B-doped g-C_3_N_4_ on the parallel plane. (The red areas represent a high probability of electrons, while the blue areas represent a low probability. The grey, blue, and pink spheres represent C, N, and B atoms, respectively). (Reprinted with permission from Ref [58]. Copyrights 2019 John Wiley and Sons).

For photocatalytic reactions, electrons are excited from nitrogen-p orbitals to carbon in g-C_3_N_4._ These electrons are mostly confined around N, which is also one of the active sites for the reaction. DFT studies showed that modifier elements like boron could be doped into carbon nitride to overcome the charge transfer effect. The theoretical calculation also proved that electron excitation from B to N in B-doped g-C_3_N_4_ is easier compared to N to C in pure g-C_3_N_4_ thus resulting in higher catalytic activity when B-doped g-C_3_N_4_ is used as a catalyst (Figure 5) [59].

#### 2.1.3. Temperature

Temperature affects the CO_2_ solubility in water. The more the quantity of dissolved CO_2_ in water, the better will be the product efficiency. A nearly 2.5 times increase in CO_2_ solubility (in water) was reported as the water temperature decreased from 25° C to 0° C. Besides solubility, lower temperature also affects the ease with which the reactants adsorb on the catalyst’s surface. This is due to lower thermal agitation, which allows easy adsorption of reactants on the catalyst surface, thereby increasing the photocatalytic reaction rate. Consequently, temperature also affects the desorption rate. When the temperature is low, the reactive intermediates and end products are more likely to remain adsorbed on the catalyst surface, inhibiting catalysis or producing catalytic poisoning, thereby reducing photocatalytic CO_2_ reduction. The lower temperature also decreases the diffusion rates and collision frequencies, affecting the product formation [60]. Thus, considering collision rate, solubility, desorption, and other parameters as key factors for CO_2_ reduction, an optimum temperature needs to be maintained.

#### 2.1.4. Size of photocatalyst

Generally, reducing the size of semiconductors increases the surface-to-volume ratio and exposes a significant number of atoms to reactant molecules (CO_2_ and H_2_O). When the size approaches the quantum dot scale, due to the strong confinement of electrons, the separation between CB and VB increases (widening of the band gap) [61]. This wider bandgap can result in a more positive VB position or a more negative CB potential. Either way, the oxidative or reductive ability of the photogenerated charge carriers increases. The larger difference between the reduction potential of the conduction band and the reduction potential of the specific reaction kinetically improves the reaction rates due to the stronger driving force of charge carriers [62]. Furthermore, reducing the size of the catalyst to the nanoscale enhances the catalyst surface area, which in-turn provides a larger number of active sites for the reactant species.

#### 2.1.5. pH

The solvent pH is an important factor affecting the end product by altering the theoretical CO_2_ reduction potential due to different proton concentrations. In acidic pH, due to high proton concentration, the theoretical CO_2_ reduction potential is lowered, favourably reducing CO_2_ to CH_3_OH. However, if the proton concentration is higher (lower pH), the condition could boost H_2_ production by stimulating H_2_O splitting compared to CO_2_ conversion [63]. However, Omadoko et al. reported enhanced formate production in an acidic medium due to an increased proton concentration [64]. When it comes to alkaline pH, contradictory results were reported. CO_2_ solubilizes very well in an alkaline medium but produces carbonates and bicarbonates, which are difficult to reduce further. These negatively charged carbonates and bicarbonates act as hole scavengers by donating electrons to the photocatalyst. The whole process results in oxygen evolution from hydroxides rather than reduction of CO_2_ [65]. To confirm the hypothesis, CO_2_ reduction studies were carried out in an alkaline medium using NaOH, and traces of CO were obtained, but no traces of methanol, formic acid, and other products were detected [66]. These results indicate that the formation of intermediates plays a vital role in product formation. These intermediates are formed dominantly in selective pH conditions [67]. Electrochemical CO_2_ reduction using gold gas diffusion electrodes (GDE) in an acidic medium was recently reported. In an acidic medium, the protons, to some extent, neutralise the OH- formed during the reaction. In an alkaline medium, CO_2_ itself neutralizes the OH-, thus lowering the reactant concentration at the reaction interface. Also, the acidic medium produces fewer bicarbonates; hence, the carbon and efficiency loss are negligible [68]. To overcome pH-related hurdles, researchers prefer gas-phase reactions where water vapor is injected as a proton donor into the photoreactor instead of an aqueous system [69].

#### 2.1.6. Pressure

The conversion of CO_2_ into useful products is also governed by CO_2_ gas pressure. Under pressure, CO_2_ solubility in water increases which facilitates a subsequent increase in the rate of reaction/product formation. Also, increasing pressure for effective CO_2_ reduction is preferred over cooling down the reactor because the solvent may show a slower desorption rate at a lower temperature, clog the active sites, and eventually decrease the reduction process [60]. According to the Langmuir Hinshelwood Model, partial pressures of CO_2_ and H_2_O are likely to impact the reaction rates [70].

#### 2.1.7. Light irradiation setting

The intensity of incident light determines the kinetics of the photo-assisted CO_2_ reduction reaction, and the fraction of the light is absorbed by the photocatalyst, which aids in system design. Incident light plays a vital role in accounting for the amount of light absorbed by the photocatalyst and media to generate desired reactant intermediates. In order to achieve better light harvesting, incident radiation must have some threshold energy greater than the band gap of the catalyst for easy transfer of electrons from VB to CB. Very high intensity can cause saturation of photocatalysts. It becomes difficult to account for light transport in the case of non-differential photoreactors. In such cases, the position of the light source and photocatalyst and the distance between them are studied to understand the influence of intensity and light transport on the rate of reaction [71,72].

#### 2.1.8. Catalyst loading/concentration

For CO_2_ reduction, photocatalyst can be loaded in two steps: 1) initial loading, where all the catalysts are introduced before the experiment, and 2) stepwise loading, where an equal amount of photocatalyst is introduced at some intervals of time. The second one yielded more product after some time. In contrast, the initial loading step showed a higher yield during the first 6 h of the experiment and later showed saturation in methanol yield. It is quite explanatory that in the initial loading process, more active sites are detected by CO_2,_ leading to early saturation. This step was also found to undergo rapid catalyst poisoning. In the case of stepwise loading, progressive growth in the product was obtained due to the addition of fresh catalyst with a higher yield compared to the initial loading process [60]. The adsorption of CO_2_ and H_2_O is said to have a direct relationship with catalyst concentration. If the concentration is low, there may be a deficiency of active sites on the catalyst, whereas a higher concentration can be ascribed to the light scattering effect, which prevents proper irradiation in the entire catalyst volume [73].

### 2.2. CO_2_ adsorption modes and conversion pathways

**Figure 6.**
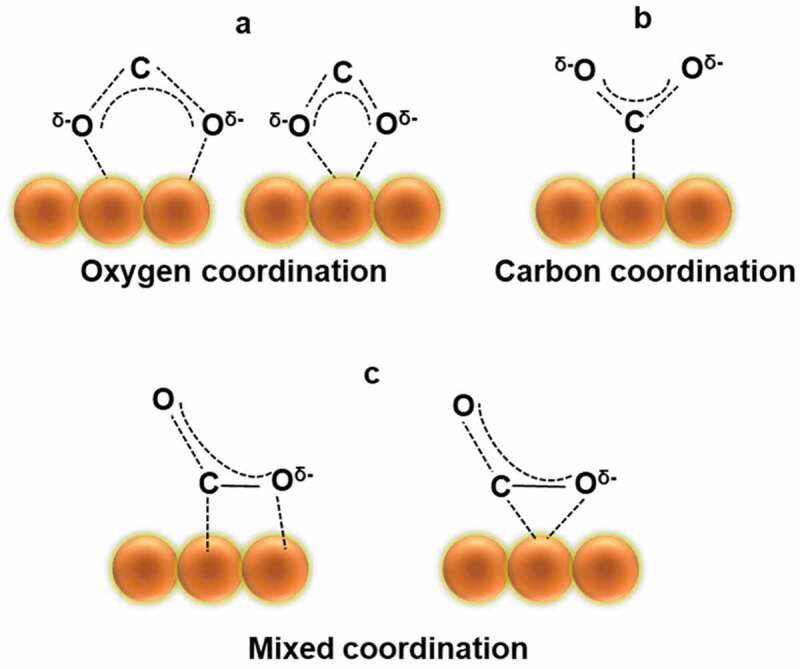
Possible structures of CO_2_ anchoring on the surface of the catalyst. (Reprinted with permission from Ref [74]. Copyrights 2006 Elsevier).

**Figure 7.**
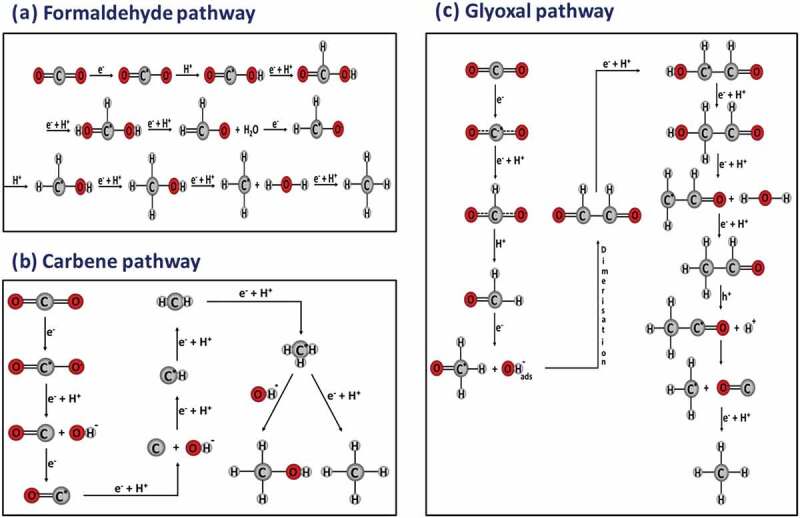
Stepwise CO_2_ reduction mechanism with (A) formaldehyde pathway, (B) carbene pathway and (C) glyoxal pathway. source [75].

CO_2_ activation and conversion involve as many as 8 electrons/8 protons and cleavage of C=O bond to form C-H bonds. These electrons and protons are responsible for generating intermediates and eventually the end product. However, as stated earlier, due to the high stability of CO_2_ and linear structure, the rate-determining step in the entire process is CO_2_ adsorption. There are three types of CO_2_˙ˉ adsorption modes reported on the catalyst surface. Each binding mode leads to a unique CO_2_ reduction pathway (mechanism) and produces a wide range of products with different yields and selectivity. Some of the binding modes (Figure 6) with probable mechanisms for converting CO_2_ to CO and COOH are discussed. The formed CO and COOH can further undergo hydrogenation or reduction in the presence of electrons to obtain the desired product. When oxygen is coordinated with the catalyst (Figure 7(a)), the carbon of CO_2_ combines with hydrogen forming intermediates (reaction below, Equation (1)) and formic acid as the end product [74]. Further, formic acid can be reduced or hydrolysed into various hydrocarbons. If carbon is bound to the surface (Figure 7(b,c)), hydrogenation becomes difficult. Hence, hydrogen attacks one of the oxygen in CO_2_˙ˉ to form a carboxyl radical, which combines with hydrogen to form formic acid. However, if the hydrogen atom is attached to the oxygen atom in CO_2_˙ˉ, an intermediate cleaved bond will be formed between O and C atoms to produce CO [76]. The formed CO can easily undergo desorption from the surface of the catalyst due to weak adsorption properties. Thus, by tailoring the catalyst surface for the desired CO_2_ binding mode, the product distribution of CO_2_ photoreduction may be regulated [77]. The formation of intermediates on the surface of a transition metal like Cu [74] and Ni [76,78] was also reported.

(1) $$\dot{C}{\overset{ˉ}{O}}_{2}+H^{+}\overset{\;}{⟶}\dot{C}OOH$$

(2) $$\dot{C}OOH+e^{-}\overset{\;}{⟶}HCOO^{-}$$

(3) $$HCOO^{-}+H^{+}\overset{\;}{⟶}HCOOH$$

The electrons in the photocatalyst should possess a more negative chemical potential for CO_2_ reduction, whereas, for water oxidation reaction, holes should be on a greater positive potential level. The CO_2_ anion radical (CO_2_-) is formed by the direct reduction of CO_2_ when one electron is transferred to the antibonding orbital of CO_2_. This procedure, however, demands a negative redox potential of −1.97 V versus SHE in an aprotic solvent (e.g. N, N-dimethylformamide) and −1.90 V in water (pH 7), which is extremely unfavourable. The proton-assisted multiple electron transfer process is a kinetically and thermodynamically favourable process that requires less energy. After CO_2_ activation by single-electron transfer, the reaction proceeds via a series of fundamental steps that include radical formation, proton and electron transfer, breaking the C-O bond, and forming C-H bonds. During this process, the recombination rates of different intermediates may account for different pathways. The difference between both paths is in the CO_2_˙ˉ binding approach (Figure 6) on the catalyst surface. Some of the possible pathways (Figure 7) are discussed below:

#### 2.2.1. Formaldehyde pathway

This pathway involves the formation of formaldehyde as an intermediate in order to convert CO_2_ to CH_4_ without involving CO. In this pathway, the formation of the carboxyl radical (˙COOH) is favored by the unidentate binding of oxygen on the surface of the catalyst or binding through carbon which is O bridged on the surface [74]. This carboxyl radical further combines with hydrogen radicals and an electron, producing formic acid. The reaction prefers the formaldehyde pathway in an aqueous medium with a high dielectric constant. The formic acid further accepts one ˙H to form a dihydroxy methyl radical, which undergoes dehydration when one more ˙H is added and forms the formaldehyde (Figure 7(a)). Formaldehyde in the presence of two eˉ undergoes reduction to form methanol and further reduction of methanol in the presence of two more eˉ produces methane. In the process, formaldehyde and methanol are intermediates and not side products [75].

The carbene pathway contains CO as an intermediate, along with methanol and formaldehyde. When CO_2_˙ˉ is linked to the catalyst via the carbon atom, the carbene pathway is preferred. The attachment of ˙H to the O of CO_2_˙ˉ causes the breaking of the C-O bond. The CO left on the surface can accept 2 additional electrons leaving carbon residues on the surface. These radicals can further combine with 4˙H to form a CH˙ radical, carbene, a methyl radical, and ultimately methane. In case, if methyl radical combines with OH radical, methanol is formed [62]. This methanol is an intermediate rather than a side product, and no traces of HCHO were found. Carbene pathway on isolated Ti^4+^ species embedded on the zeolite surface has been reported. Here, the quantum confinement effect resulted in the formation of charged transfer excited state species (Ti^3+^O)*where photogenerated electrons and holes are localized on the neighboring atom. If a metal co-catalyst is not used, then this charge transfer excited state species (Ti^3+^-O-)* forms methanol as a major product [79].

#### 2.2.2. Glyoxal pathway

The electron paramagnetic resonance spectroscopy (EPR) investigation of CO_2_ reduction intermediates such as formyl radicals (HC˙O), which dimerize to form glyoxal, led to the proposal of the glyoxal pathway [80]. The reaction involves reduction as well as oxidation (Figure 7(c)) following the sequence CO_2_˙ˉ → HC˙O → HOCCOH → HOCH_2_COH → HOCCH_2_˙ → HOCCH_3_ → CH_3_OC˙ → CH_3_˙ → CH_4_. The formation of CH_4_ from C_2_ intermediates, like CH_3_COOH and CH_3_CHO, has been reported over the TiO_2_ surface [81]. Although this mechanism could not rationalize the formation of formic acid or methanol, the prominent feature of predicting an equal ratio of CH_4_ and CO in the experiment with TiO_2_ anatase was reported [82].

From the above discussion, it is quite evident that the pathways and product selectivity rely on the anchoring of CO_2_˙ˉ on the catalyst surface [76], followed by electron/proton transfer. A recent modification was reported where the reaction proceeds via the formaldehyde pathway CO_2_˙ˉ → CO → HCHO → CH_3_OH → CH_4_ on an ideal surface through a modified pathway CO_2_˙ˉ → CO → HCHO → CH_3_˙ → CH_4_/CH_3_OH, combining both formaldehyde and carbene mechanism on the surface with oxygen vacancies [83].

In all these three pathways, the formation of methoxy (.CH) intermediate remains an important step in CO_2_ photocatalytic reaction. Depending on the byproduct, hydroxyl radical (OH.) or H+ may combine with methoxy intermediate to form CH_4_ or CH_3_OH. The presence of unpaired electrons in these radicals allows us to study the reaction mechanism in depth via EPR. However, detailed research needs to be done to understand the reason behind the broad range of product selectivity and accurately predict the final products in case of CO_2_ photoreduction.

## 3. Factors affecting hydrogen evolution

**Figure 8.**
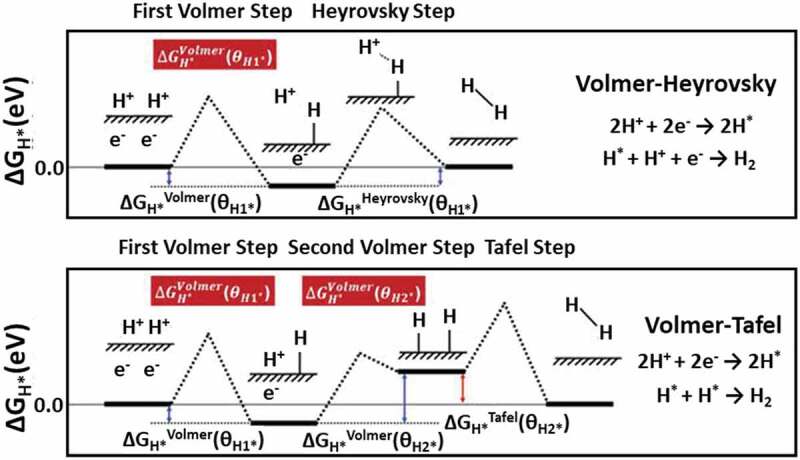
Schematic of Volmer-Heyrovsky and Volmer-Tafel pathways for HER process. Source [84]: .

The hydrogen evolution reaction (HER) acts as a counter half-reaction to the oxygen evolution reaction (ORR) involving a two-electron transfer process (2 H+ +2e- → H_2_) that offers hydrogen production. During the electrolysis process, HER requisites have to be at the same kinetics as a counter to OER to maintain equilibrium. The H_2_ production from the water proceeds through different routes like the Volmer-Heyrovsky or Volmer-Tafel pathways as presented in Figure 8. Under the alkaline condition, initially during Volmer-step, hydrogen intermediates H_ad_ (H_2_O + e- + catalyst = catalyst-H_ad_ + OH-) are formed; thereafter, the Heyrovsky step (H_2_O + catalyst-H_ad_ + e- = catalyst + H_2_ + OH-) or combined Tafel step (2 catalyst-H_ad_ = catalyst + H_2_) arises. In acidic conditions, the other steps are similar except for the hydrogen intermediate formation that includes the discharge of H ions [85]. Splitting of water is a non-spontaneous reaction, thermodynamically, and to precede the water decomposition process at an appreciable rate, excess energy is required as ΔG > 0. Electrochemical water splitting reports several kinetic as well as thermodynamic barriers as the process results in energy loss during the reaction giving rise to higher overpotential of the HER and OER. To achieve higher efficiency, the catalyst is necessary to have a low overpotential. As reported, Pt is the top known catalyst for HER with the need for the least overpotential due to high intrinsic electrocatalytic activity and can meet the industrial demand but at a higher material cost. To reduce the price of H_2_ production, it is important to either boost Pt as a noble metal-based hybrid alloy or to go with some non-noble metal-based alternatives [85]. Another method, like downsizing the size of the particle, can utilize the material more effectively, and the efficiency of the electrocatalyst should be improved.

A number of materials have been investigated for better adsorption of CO_2_ and hydrogen gas production. To eliminate the reported drawbacks, factors such as higher efficiency, synthesis cost, half-life, and easy fabrication were also taken into consideration. Meanwhile, ample attention is being given to the photocatalytic route for the reduction of CO_2_ and photocatalyst-based water splitting for HER. This review summarises the work done in this field for the development of the catalyst along with CO_2_ reduction. The performance of different chalcogenides, metal organic framework, and single-atom catalysts as an example of ultimate size reduction up to atomic level is reviewed.

## 4. TiO_2_ based materials as the catalysts

TiO_2_ is a very popular photocatalyst due to properties like a low price, high stability, non-toxic nature and high efficiency in photocatalytic reactions [86]. TiO_2_ occurs in three different allotropes, namely anatase, rutile, and brookite, showing different photocatalytic activity as the phase changes. Anatase is metastable and said to have high photocatalytic activity, whereas rutile with lower photocatalytic activity inhibits good stability. It was observed that TiO_2_ containing a mixture of both rutile and anatase phases exhibited a synergistic effect, resulting in higher photocatalytic activity than their individual phase [87,88]. However, TiO_2_ inherits certain drawbacks as a photocatalyst material, such as:

- The number of photogenerated holes and electrons coexisting in TiO_2_ leads to higher recombination rates and lower chemical activity with respect to absorbed light [89].
- The wide bandgap of TiO_2_ (3.2 eV) allows absorption of a UV component that holds only 4% to 5% of the share of the total solar spectrum [90,91]
- Crystalline TiO_2_ is mostly non-porous in nature with a polar surface, thus decreasing the absorption activity of non-polar organic solvents [92].

Several strategies are being developed to overcome these drawbacks and increase the activity of TiO_2_ for photocatalytic CO_2_ reduction [76,90,91,93]. Sarkar et al. synthesized TiO_2_ nanofibers (NFs) and studied the effect of noble metals (Pt and Pd) on CdSe QD, which served as co-catalyst. Although the addition of these co-catalysts increased the reaction rate, Pd containing TiO_2_ NFs showed superior methanol selectivity than Pt-TiO_2_ NFs. When CdSe QD is anchored with 1% Pt decorated TiO_2_ NFs, methanol yield of 90.22 ppm/g/h and 225.4 ppm/g/h formic acid was recorded, whereas a drastic decrease in product formation was observed when CdSe QD was anchored with Pd decorated TiO_2_ NFs (4.63 ppm/g/h of methanol and 13.11 ppm/g/h formic acid). The authors offered credit to Pd nanoparticles for the decomposition of CdSe QDs, which decreased the yield of methanol [94]. Ikeue et al. synthesized Ti-zeolites in various conditions, employing the OH- and F-ions as structure-directing agents, and investigated their CO_2_ reduction ability in an aqueous medium. The authors studied the difference in water affinity between Ti-β(OH) [hydrophilic] and Ti-β(F) [hydrophobic] and how it affects the reactivity and selectivity for photocatalytic CO_2_ reduction in the presence of H_2_O. Methanol selectivity on the surface of Ti-β(OH) was 41% greater than Ti-β(F), which was credited to the larger concentration of charge-transfer excited complexes of Ti-β(OH) [95]. Anpo et al. reported artificial photosynthesis on single-site Ti photocatalyst constructed with various zeolite frameworks. They also reported that TiO_4_ unit single-site catalysts embedded within the framework of several porous materials such as zeolites and mesoporous molecular sieves exhibited unique photocatalytic reactivity as compared with normal TiO_2_ photocatalysts [96]. The authors claimed that photo-generated charge carriers are present on the same site, such as a pair of Ti^3+^ and O- within the TiO_4_ single-site catalysts that work together to give high reactivity. It was found that the large surface area, shape selectivity, and molecular diffusion properties of the zeolite framework enhanced the charge transfer effect and the modified structure due to metal ion implantation/doping resulted in the absorption of visible light [96].

The poor solubility of CO_2_ is another reason for the low product formation. Tseng et al. addressed this issue by simply adding a small amount of NaOH into the photocatalytic reactor. This caustic solution dissolved more CO_2_ as compared with water which led to increased methanol yield over Cu doped TiO_2_ photocatalyst. CO_2_ being chemically acidic, undergoes better solubility in the presence of base, thus affecting the overall product formation. Furthermore, the OH- in the solution acts as a powerful hole scavenger that improves the charge separation [97]. Another factor responsible for the poor efficacy of the CO_2_ photoreduction process is the recombination of photogenerated charge carriers before they get transferred to active sites. To overcome this, the photocatalyst with a lower work function is doped with metals of a higher work function to facilitate electron capture. This enhances the ability of the metals to accept the photogenerated charge carriers and reduces the recombination chances. The overall effect is the improvement in the reductive performance of the supported metal catalyst [98]. Michaelson et al. calculated the work function of different metals [99] and arranged them in order; Ag/TiO_2_< Au/TiO_2_< Pd/TiO_2_< Pt/TiO_2_ with platinum having a work function of (5.93 eV), palladium (5.60 eV), gold (5.47 eV), and silver (4.74 eV) [100]. Xie et al. doped noble metal cocatalysts and checked the doping effects on the product selectivity. The rate of CH_4_ production increased in the order; Pt > Pd > Au > Rh > Ag, indicating an improvement in the charge separation efficiency. This study also reported the importance of co-catalyst on product selectivity, which is enhanced to 83% in the presence of Pt compared to bare TiO_2,_ which is 24% [101].

**Figure 9.**
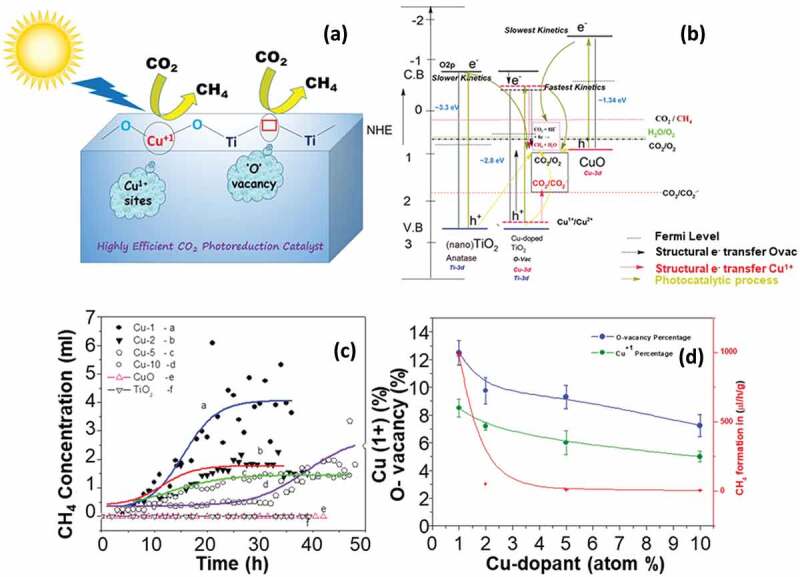
(a) Scheme highlighting the role of Cu^+1^ sites and oxygen vacancies in CO_2_ photoreduction; (b) Schematic for the photocatalytic and the structural adsorption processes occurring with TiO_2_ anatase, Cu-doped TiO_2_ (with presence of oxidation state Cu^1+^) and CuO; (c) Plot for the CH_4_ concentration with respect to the irradiation time for a) Cu-1; b) Cu-2; c) Cu-5; d) Cu-10; e) CuO f) TiO_2_ nano; (d) Photocatalytic methane production as a function of Cu^1+^ sites (%) and O-vacancy sites (%) in Cu-doped TiO_2_ samples (Reprinted with permission from Ref [69]. Copyrights 2021 the journal of physical chemistry C).

In recent years, Cu has emerged as a standard co-catalyst for highly selective CO_2_ photoreduction to CH_4_ and CH_3_OH. The synthesis of Cu-doped TiO_2_ photocatalysts (Cu, 1–10%) and their CO_2_ photoreduction activity in the presence of water vapor have been reported [69]. (Figure 9(c,d)) indicate that the lowest doping (Cu – 1%) showed the highest selectivity and maximum CH_4_ generation (980 μL/g/h). The authors also determined via X-ray photoelectron spectroscopy (XPS) and (EPR) spectroscopy that the clustering of Cu^2+^ states at higher doping concentrations (Cu-10%) is unfavourable to photoactivity and that more electronegative surface [Cu^+1^ sites], as well as oxygen vacancies, play an important role towards CO_2_ activation and photoreduction (Figure 9(a,b)). XPS examination of the used catalyst surface revealed the absence of the -OH groups and reduction in the Cu^1+^ sites, which revealed the effective utilization of these sites. It was proposed that the transfer of electron to the CO_2_ antibonding orbital mostly occurs through the doped Cu sites, as represented in the scheme (Figure 9(b)). The presence of Cu^1+^ sites facilitates the kinetics of the photocatalytic electron transfer that leads to more CH_4_ yield in the case of the Cu-1 sample as compared to Cu-10 sample. As shown in the scheme (Figure 9(b)), CuO slows down the rate of electron transfer (kinetic) and undesirably affects the initiation of the CO_2_ adsorption. Therefore, the observed CuO cluster on the surface of Cu-10 sample made it a less photoactive material due to the reduction in Cu^1+^ and O-vacancies. Lower surface area also limits the efficiency of the photocatalyst. There are two straightforward ways to increase the surface area: (1) decreasing the size of the photocatalyst to the nanometer scale and (2) synthesizing nanoporous photocatalysts. In the first approach, size reduction means a finite number of atoms form the particle as compared with its bulk counterpart. When the size is further trimmed down to the nanoscale, the confinement of electrons leads to the quantization of their energy and momentum. It causes less overlapping of atomic energy levels and as a result, the band gap increases. This band-gap engineering for tuning the light absorption properties of the catalyst is also known as the quantum confinement effect. Along with band gap tuning, decreasing the catalyst size not only offers a higher surface area that uncovers a large number of active sites for CO_2_ adsorption but also provides a shorter path length for charge carriers [102]. Low et al. investigated the quantum confinement effect and found that nanoparticles with smaller diameters had a larger band gap energy, which prevents the migration of electrons from the CB of TiO_2_ to Pt. Therefore, engineering the size of the nanoparticle is important for the rapid migration of photogenerated electrons from TiO_2_ to Pt NPs [100]. Li et al. produced mesoporous silica-supported Cu/TiO_2_ and carried out CO_2_ photoreduction in a continuous flow reactor to boost photocatalytic activity. The authors attributed the increase in the CO_2_ reduction efficiency to the larger surface area of mesoporous silica substrate (>300 m^2^/g) that provided high dispersion of TiO_2_ sites and also upgraded the adsorption of CO_2_ and H_2_O [103]. Apart from metals [104] and non-metals [105], lanthanides [106] were also impregnated into the TiO_2_ lattice which indicated improvement in the CO_2_ photoreduction due to the synergistic effect between the catalyst and the dopant.

Gao et al. performed water splitting over an Mg-doped TiO_2_ photocatalyst. The synthesized Mg-doped TiO_2_ was found to eliminate the deep defect states near the valence band and minimize the defect states located under CB in TiO_2_. Here, the 2p orbitals of Mg could hybrid with the intrinsic defect states induced by oxygen vacancy and place those defect states out of the bandgap, thus affecting the overall photocatalytic water splitting [107]. Similar to CO_2_ photoreduction, coupling TiO_2_ with noble metals has been discovered to exhibit a major role in accelerating charge transfer and is regarded as a useful technique to improve HER efficiency. Kamat et al. demonstrated the effectiveness of the [TiO_2_-noble metal] system for photocatalytic reaction. When Au colloids make direct contact with TiO_2_ nanoparticles in the reaction medium, electrons are transported from TiO_2_ to Au, which causes a Fermi-level shift. The improved charge separation driven by this shift is beneficial for enhancing the efficacy of photocatalytic processes [108]. Ortiz et al. synthesized Ag-doped TiO_2_ (named-TiO_2_Ag-F) using sol-gel/solvothermal (SGH) treatment and another sample (named-TiO_2_Ag-C) by using sol-gel/solvothermal/thermal (SGHT) technique. Here, the results revealed that the size of TiO_2_ crystals decreased in the presence of elemental Ag, which serves as a physical barrier between TiO_2_ crystals to prevent anatase crystal formation during solvothermal treatment. The surface plasmon resonance (465 nm) of Ag nanoparticles and the availability of the energy states above VB resulted in decreasing the energy bandgap from 3.05 eV (anatase) to 2.8 eV in TiO_2_Ag-F and 2.6 eV in TiO_2_Ag-C. When irradiated under visible light for 4 h, TiO_2_Ag-F showed 180 µmol/g of H_2_ production [109]. Considering efficiency, cost, and robustness, TiO_2_ and its modified versions are benchmark photocatalysts for both CO_2_ reduction and H_2_ evolution.

**Figure 10.**
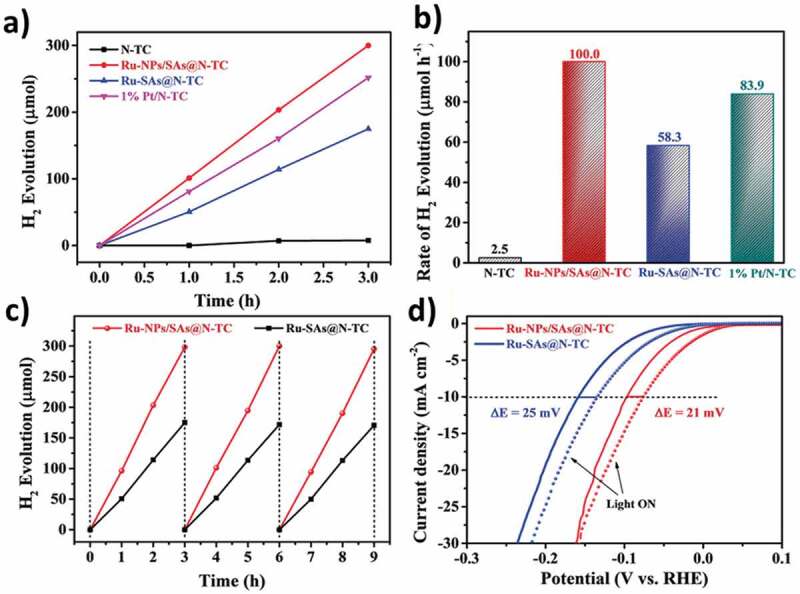
(a) Time-dependent photocatalytic H_2_ production and (b) the corresponding rates of H_2_ evolution over N-TC, Ru-NPs/SAs@N-TC, Ru-SAs@N-TC, and 1% Pt/N-TC under 300 W Xe lamp, *λ* = 320–780 nm (20 mg of catalyst dispersed in 100 mL of a mixed solution of water and methanol with v/v = 4:1). (c) Photostability of H_2_ evolution and (d) photo-assisted electrocatalytic LSV polarization curves of Ru-NPs/SAs@N-TC and Ru-SAs@N-TC in 1 m KOH. (Reprinted with permission from Ref [110]. Copyrights 2020 John Wiley and sons).

Recently, Yan et al. performed photocatalytic/electrocatalytic HER studies on N-doped TiO_2_/C support, derived from the amine-functionalized metal-organic framework (NH_2_-MIL-125). The authors were able to include two types of Ru species, nanoparticles (NPs) and/or single atoms (SAs), using the stabilizing effect of surface NH_2_ groups and pore confinement, and obtained two types of samples, Ru-NPs/SAs@N-TC and Ru-SAs@N-TC, respectively. Ru-NPs/SAs@N-TC showed superior H_2_ production and a decrease in overpotential (Figure 10), producing 100 µmol/h than Ru-SA@N-TC (58.3 µmol/h) and even 1% Pt/N-TC samples (83.9 µmol/h), under visible light irradiation. The authors attributed the improved catalytic activity of Ru-NPs/SAs@N-TC to the synergistic interaction of Ru NPs and SAs [110]. Very recently, the synthesis of TiO_2_ nanosheet-carbon composites was obtained from MXene precursor by hydrothermally treating Ti_3_C_2_T_x_ (T = OH, F, and O) at 200° C followed by annealing at 500° C. The obtained catalyst showed 69 µmol/g/h H_2_ evolution under visible light irradiation as compared with commercial P25 particles. Although the approach is new, the requirement of very high temperature (1450° C) for Ti_3_AlC_2_ preparation, the use of HF for etching to get Ti_3_C_2_T_x_ and the low rate of H_2_ production brings serious limitations [111].

Defect engineering is a very useful strategy to achieve visible light harvesting in TiO_2._ However, defects like oxygen vacancies mostly serve as recombination traps and obstruct the charge separation and transport process. To address this issue, Hu et al. took advantage of the interaction between metal ions and electrons confined at O-vacancies to dope noble metal [Pt, Au, Ag] NPs on defective TiO_2_ hierarchical spheres (THS). Commercial TiO_2_ (C-TiO_2_) cannot absorb visible light and therefore produces a negligible quantity of H_2_ while C-TiO_2_-Pt showed very weak photocatalytic activity under visible light irradiation (0.07 mmol/g^−1^h^−1^). Defect-rich THS sample reported very low activity (0.02 mmol h^1^ g^1^), indicating the recombination due to O-vacancies. Under only visible light irradiation, the largest rate of hydrogen evolution was attained by THS-Au (1.49 mmol/h/g) and not by THS-Pt (1.06 mmol/h/g), while the THS-Ag exhibited the lowest rate (0.44 mmol/h/g) [112]. Interestingly, THS-Au, THS-Ag, and THS-Pt, when exposed to UV and visible light, delivered 8.06, 3.29, and 13.16 mmol/h/g of hydrogen evolution rate, respectively, much more than THS.

Wu et al. studied the effects of doping TiO_2_ nanofibers with 11 transition metals [Ag, Au, Co, Cr, Fe, Cu, Y, Ni, Pt, Pd, and Zn] separately for photocatalytic hydrogen production. The Cu-doped TiO_2_ sample showed a maximum yield of 200 μmol/h/g under UV-A light irradiation and 280 μmol/h/g under UV-B light exposure [113]. The density of states calculated by CASTEP indicates that Cu doping introduces the states near the VB edge and decreases the bandgap. Mor et al. prepared a p-n junction photochemical diode by studying different orientations of Cu-Ti-O (p-type) nanotube films in combination with TiO_2_ (n-type) nanotube array films. The system showed very good performance towards hydrogen generation by water splitting with photocurrents of 0.25 mA/cm^2^, and photoconversion efficiency of 0.30% [114]. While the TiO_2_ surface is still being modified with various techniques to narrow down the bandgap for visible light absorption, researchers are also working on developing non-TiO_2_ photocatalysts. Synthesis of various non-TiO_2_ nanomaterials, their doping techniques, and photocatalytic activities was well documented in the literature [94,95,98,103–106].

## 5. Non-TiO_2_ materials

This section summarizes the performance of efficient non-TiO_2_-based materials for photocatalytic reactions. Wu et al. in their recent review compiled research on developing hybrid semiconductors and fundamentals of electrochemical and photochemical reactions [115]. They also classified catalysts into three different groups and discussed their product selectivity. As discussed earlier, CO_2_ adsorption/capture is an energy-demanding step in the CO_2_ reduction process. Considering this, Luo et al. synthesized functionalized ionic liquid (ILs), mainly by introducing the nitrogen-based interacting sites on the imidazolate anion and phenolates to improve the CO_2_ adsorption. The multiple-site interaction (N and O) between the pyridine containing ILs and CO_2_ led to the larger CO_2_ capture capacity (1.60 mol CO_2_ per mol/L) and very good reversibility when pressure is reduced [116]. Single crystalline zinc orthogermanate (Zn_2_GeO_4_) nanoribbons with a very high aspect ratio (10000) were produced by the solvothermal technique for effective CO_2_ reduction. This material produced 1.5 µmol g^−1^ of CH_4_ during the first hour under light illumination and the rate of CH_4_ generation was substantially enhanced by loading Pt, RuO_2_, or co-loading Pt and RuO_2_ as a cocatalyst [117]. Yu et al. synthesized a composite structure of reduced graphene oxide (RGO) and CdS nanorods, which exhibited a higher photo-conversion of CO_2_ to CH_4,_ even in the absence of a noble metal cocatalyst. Controlled experiments made it clear that the absence of RGO hampered the production rate (0.21 mmol/h/g) of CH_4,_ which increased (2.51 mmol/h/g) on the surface of RGO-CdS nanorods with optimum Cu wt.% of 0.5. The lower photocatalytic activity was attributed to a higher recombination rate on the CdS surface, which eventually decreased when RGO was introduced [118]. Furthermore, Cu_2_O/RGO composite reported by An et al. showed 50 times higher CO_2_ photoreduction activity than Cu_2_O alone after 20 h irradiation. This finding confirmed that RGO coating gives stability to Cu_2_O, retards recombination, and improves charge separation and transport mechanism [119]. The following sections will focus majorly on the performance of non-TiO_2_-based materials towards CO_2_ photoreduction and HER.

### 5.1. Metal chalcogenide catalysts

**Figure 11.**
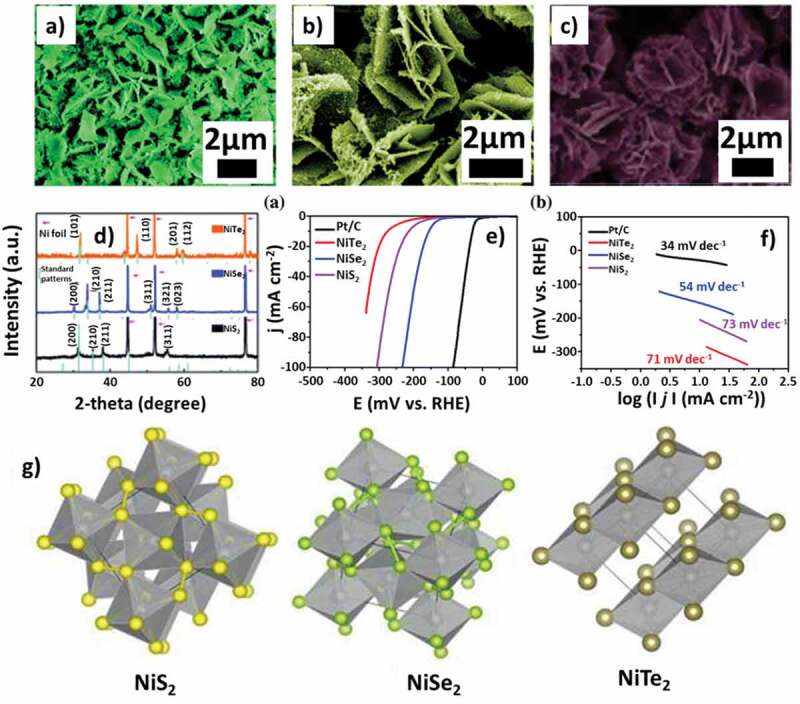
(a, b, c) SEM image of NiTe_2_, NiSe_2_, NiS_2_ respectively, (d) XRD pattern of NiTe_2_, NiSe_2_, NiS_2_, (e) LSV curve for HER measurement in 0.5 M H_2_SO_4_, (f) Tafel slope of materials, (g) Crystal structure of Nickel chalcogenides NiS_2_, NiSe_2_ and NiTe_2_ *Source*: [125].

Chalcogenides are a promising alternative for HER in electrocatalytic activities because of their high chemical stability and good conductivity. Several metal chalcogenides showed better catalytic activity than TiO_2_ based materials. Transition metals like Ni, Co, and Fe could bond with sulfides, selenides, and tellurides and form different chalcogenides. As per the previous results, Ni-based chalcogenides are the best in performing material as an electrocatalyst (Table 2). Ouyang et al. [120] designed an array of Ni_3_S_2_ nanorods on AT-Ni foam that displayed 200 mV of overpotential for a current density of 10 mA cm^−2^ which was comparatively better than the other non-noble-based metal catalysts, reported earlier. The reason behind this performance was the presence of fast electron transfer channels in Ni_3_S_2_ nanorods. This structure also provides access to better active site nanoparticles such as NiS, NiS_2_, Ni_3_S_2_ through the facile microwave-assisted solvothermal method. The highest performance of Ni_3_S_2_ can be due to its intrinsic conductivity. Chung et al. [121] prepared nickel sulfide nano-electrocatalyst material and observed a better overpotential of 88 mV (10 mA/cm^2^ current density) than 117 mV for Ni_3_S_2_. They confirmed the higher activity of NiS because of their strong affinity for hydrogen. Kukunuri et al. [122] developed three morphologies as spherical, hexagonal, and wires of semiconducting NiSe through the hydrothermal method. Due to its one-dimensional nature, the wire structure provides a better electron transport channel and large surface area, and therefore, it shows better activity than other morphologies. Tang et al. [123] developed a nano-wall film of NiSe_2_ from Ni(OH)_2_ using carbon cloth support via topotactic transformation with an anion exchange reaction. NiSe_2_ showed an overpotential of 145 mV (at 10 mA/cm^2^) and retained its catalytic activity for 40 h. Hydrothermal synthesis of hollow nickel telluride nanosheets was carried out [124] by using an anion exchange reaction, which showed enhanced electrocatalytic activity and stability. The ion exchange was preceded between pre-synthesized nanosheets of nickel hydroxide hexagonal structure and tellurium ions. Chia et al. [125] studied two tellurides, namely NiTe_2_ and CoTe_1.8_, and found that NiTe_2_ exhibits a lower Tafel slope (44 mV/dec) than CoTe_1.8_ (51 mV/dec) (Figure 11). Along with fast kinetics, both the tellurides displayed robust stability and improved catalytic activity. Ge et al. [126] investigated the activity and stability of nickel forming nanosheet chalcogenides with sulfide, selenide, and telluride. The authors observed an overpotential of 213, 276, 156 mV (at 10 mA/cm^2^) for NiS_2_, NiTe_2_ and NiSe_2_, respectively, that confirmed the superior performance of NiSe_2_ among the Ni-based electrocatalysts. The results were supported by density functional theory. Despite having favourable properties like narrow band gap and tunable VB/CB positions (by varying composition), metal chalcogenides lag in practical applications due to inadequate methods of synthesis and very poor chemical stability.

**Table 2.** Comparison between differently synthesized chalcogenide structures for HER. *Source*: [119],[120],[121],[122],[123],[124],[125].

Electrocatalyst	Technique	Electrolyte	Overpotential [at 10 mA cm^−2^ (mV)]	Tafel slope (mV/dec)
Ni_3_S_2_ nanorod	Hydrothermal	1 M KOH	200	107
NiS nanoparticles	Hydrothermal	0.5 M H_2_SO_4_	-	88
NiSe nanowire	Hydrothermal	0.5 M H_2_SO_4_	-	52
NiSe_2_ nanowall	Topotactic transformation with anion exchange reaction	0.5 M H_2_SO_4_	145	41
Nickel telluride nanosheets	Hydrothermal	0.5 M H_2_SO_4_	−422	87.4
NiTe_2_	Hydrothermal	0.5 M H_2_SO_4_	560	44
NiS nanosheet	Hydrothermal	0.5 M H_2_SO_4_	213	71
NiSe nanosheet	Hydrothermal	0.5 M H_2_SO_4_	156	54
NiTe nanosheet	Hydrothermal	0.5 M H_2_SO_4_	276	73

### 5.2. Metal-organic framework (MOF) based catalysts

Li et al. studied MOFs in CO_2_ reduction and discussed three main categories of MOF-based composites, namely metal-MOF, semiconductor-MOF, and photosensitizer-MOF. Higher CO_2_ yields were obtained with metal-MOFs due to the position of the lower Fermi level of metal nanoparticles as compared with the LUMO level of MOF photocatalyst, thus allowing easy transfer of electrons from MOF photocatalyst to metal nanoparticles. Alternatively, the inherent porosity of MOFs facilitates MOF-based materials to possess ultrahigh CO_2_ adsorption capacity and photocatalytic activity. The clusters of metal and the organic linkers in MOFs ease the exchange in the absorption of metal ions into MOFs. MOFs also provide several metal clusters and linker combinations that increase possibilities for the construction of new MOF photocatalysts. The same group also discussed other advantages and drawbacks of MOFs. While a range of materials were studied for the analysis of the photocatalytic process, carbon nitride (CN) with different stoichiometry also gained attention owing to their facile inexpensive synthesis, high surface area, excellent stability, non-toxic nature, visible light response, and tunable bandgap. The major concern associated with the photocatalytic performance of CN materials is the fast recombination rate of the charge carriers, and therefore, CN materials were further functionalized with other materials. Dong et al. observed and concluded that photo-reactivity was affected greatly when porosity was introduced into the g-C_3_N_4_. Photooxidation of dye increased on pg-C_3_N_4_ while photoreduction of CO_2_ on the same porous surface declined. The reason for higher photooxidation was ascribed to the surface area-dependent activity, which was enhanced due to porousification, while in the photoreduction case, there were difficulty in electronic excitation and a lot of structural defects due to porousification lower the photoreduction ability [127]. However, this judgment of decreasing activity because of the porosity may be a matter of debate based on recent reports which claim the utilization of porous nature for photoreduction of the CO_2_ [128]. Wang et al. recently reported a MOF-derived porous Cu/Zn bimetallic oxide catalyst that exhibited a very high rate (3.71 mmol/g/h) of methanol production from CO_2_ photoreduction [129]. Other materials like graphene oxide [130], sulphur [131], boron [58], carbon [132], oxides such as cobalt oxide [133], perovskite oxide [134], zinc oxide [135] and metals like potassium, lithium, sodium, rubidium [136,137], copper [138], iron [139], chromium-zinc oxide hybrid [140], platinum [141], gold [142], palladium-silver bimetal [143], palladium [144], etc., were incorporated on the CN surface of and their effects on CO_2_ reduction were also discussed in the literature.

**Figure 12.**
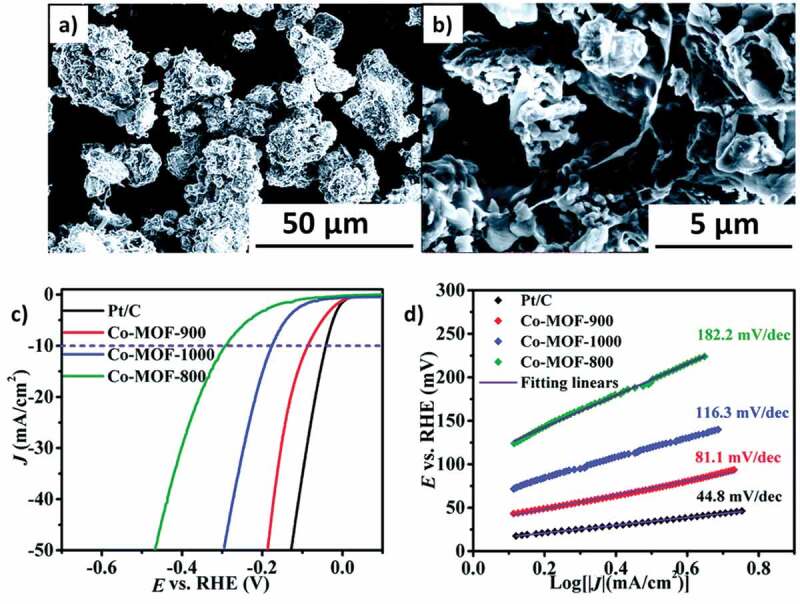
(a, b) SEM image of Co9S8@NS-C-900 composite at 50 and 5, (c) LSV curve of three different Co-MOF derived catalysts, (d) Tafel slope of different Co-MOF derived catalysts. *Source*: [148].

In the case of hydrogen production, pristine MOFs, MOF supports and MOF-derivatives-based catalysts can provide a better alternative to TiO_2_ based materials. Among these materials, MOF-derived catalysts generate heterojunction and provide effective channel charge transportation. Earlier research on photocatalytically active MOFs proved that they can achieve excellent hydrogen production through their photo-induced capability. Karuppasamy et al. [145] synthesized a MOF-derived composite NiMo/NiMoO_4_@NC with inexpensive transition metals on the nickel foam by using a hydrothermal process. This low-cost hydrogen production electrocatalyst exhibited better stability in an acidic medium. For HER, the MOF showed an overpotential of 80 mV (@10 mA cm^−2^ of current density) and exhibited an excellent activity because of their structure and merits of NiMo, satisfying the Volmer-Heyrovsky mechanism. Peters et al. [146] designed a cluster made up of a few atoms of nickel and sulfur and attached it to the Zr(IV) based MOF NU-1000 via atomic layer deposition. The obtained MOF showed excellent H_2_ production (3.1 mmol/g/h) in a buffered aqueous solution of pH 7 under UV irradiation. The authors further demonstrated that the rate of H_2_ generation could be accelerated to 4.8 mmol/g/h by adding organic dyes such as rose Bengal and NiS-AIM. Do et al. [147] designed molybdenum sulfide (MoS_x_), which had an amorphous structure by modifying Co-based MOFs through the solvothermal method. Due to this modification, the newly developed CoMoS phase could improve the activity of the MOF. The modified MOF provided a better Tafel slope of −68 mV dec^−1^ and showed good stability up to 1000 cycles. Li et al. [148] developed Co9S8@NS-C-900 composite as a novel HER electrocatalyst by pyrolyzing the Co-MOF with thiourea (Figure 12). The catalyst exhibited an overpotential of −86.4 mV and a large exchange current density of 0.40 mA cm^−2^. After 1000 cycles, there was no decay in its HER activities and the catalyst also displayed a long-term durability. The results proved that this is a noteworthy option for HER catalyst. Recently, Verma et al. reviewed the role of MOF functionalization towards CO_2_ photoreduction to CO and other important chemicals [149].

The improved activity of MOF is due to its light absorption ability, structural flexibility, well-ordered porous structure, and a huge number of available active sites. Despite several advantages like high surface area, tunable structure/composition and easy access to active sites in the porous structure, large-scale use of MOF materials is restricted due to poor selectivity, high recombination rate, low yield, high synthesis cost and low stability toward pH, temperature, and pressure (due to organic linkers).

### 5.3. Single metal atom-based catalysts

**Figure 13.**
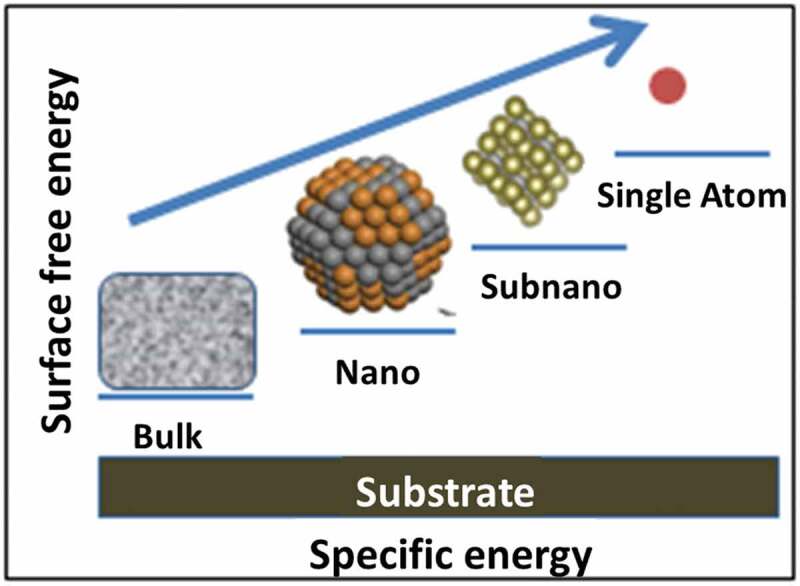
Specific energy variation with cluster size. *Source* [151].

In conventional heterogeneous catalysis, the catalytically active component is usually a metal cluster that exhibits a broad size distribution. However, a small section of the metal particles with an appropriate size distribution can serve as catalytic active species, whereas the remaining particles are either inactive or may activate unwanted side reactions. Therefore, such catalysts suffer from low efficiency (per metal atom) and poor selectivity. In recent years, a system containing atomically dispersed atoms on the solid support has attracted significant attention. This system individually distributes isolated metal atoms on high surface area/functionalized supports [150]. Single-atom catalysts (SACs) are a non-traditional heterogeneous group that offers 100% use of metal atoms with atomically dispersed metals. It is well informed in the previous literature [151] that with decreasing particle size of the metal, the specific activity per atom generally increases (Figure 13). Similarly, SACs are ultimately smaller in size and hold a significant prospect to be a highly active catalyst. Unlike nanoparticles, the structure of SACs does not remain the same and thus affects the surface free energy and specific activity and exhibits enhanced activity as compared to the conventional heterogeneous catalysts. The adsorption and the desorption capability of the active components on the SAC structure change with the size reduction, affecting the kinetics.

The latest research on supported metal cluster model catalysts showed that very different catalytic activities and selectivity could be possible when the metal centers size is trimmed to the sub-nanometer scale [152]. When the metal catalyst size is reduced to such an extent that the distribution approaches the atomic scale, the activity in certain target reactions is different compared to their nanocluster counterpart [153].

According to this approach, single metal atoms are incorporated into the surface of the solid to ensure the maximum utility of the surface atoms [154]. To improve the selectivity and efficiency of CO_2_ conversion, Zhang et al. synthesized a single atom of Co and Zn incorporated into the MOF. The results revealed the higher activity of MOF-525-Co with a CO production rate of 200.6 mmol/g/h and a CH_4_ evolution rate of 36.76 mmol/g/h under 6 h of light irradiation, which was observed to be much larger than MOF-525-Zn [CO evolution −111.7 mmol/g/h and CH_4_ yield-11.635 mmol/g/h]. In the case of undoped MOF-525, the CO and CH_4_ production rate was recorded to be 64.02 mmol g^−1^h^−1^ and 6.2 mmol g^−1^h^−1^, respectively. The major building blocks in MOFs are Porphyrin-containing struts targeting solar light harvesting and directional transport of photogenerated excitons from porphyrin to catalytically active Co sites. These porphyrin structures have exciton transport capability and display anisotropic energy propagation over tens of struts from the previously excited struts, which led to enhanced photocatalytic activity. Furthermore, doping ensures a reduction in recombination and better electron transfer. The difference in the proportion of activity enhancement between Co and Zn was credited to their different charge separation capacities as a function of the donor–acceptor interaction [155]. Huang et al. studied a single Co atom and its role on the C_3_N_4_ surface with various amounts of Co loading. The CO and H_2_ as major formed products were observed with CO_2_+@C_3_N_4,_ which was enhanced 5 times when CoCl_2_ was used as an activating agent on the same CO_2_+@C_3_N_4_ surface. A negligible amount of product was obtained on bare C_3_N_4,_ indicating the significance of a single-atom doping strategy for photocatalysis processes [156].

**Figure 14.**
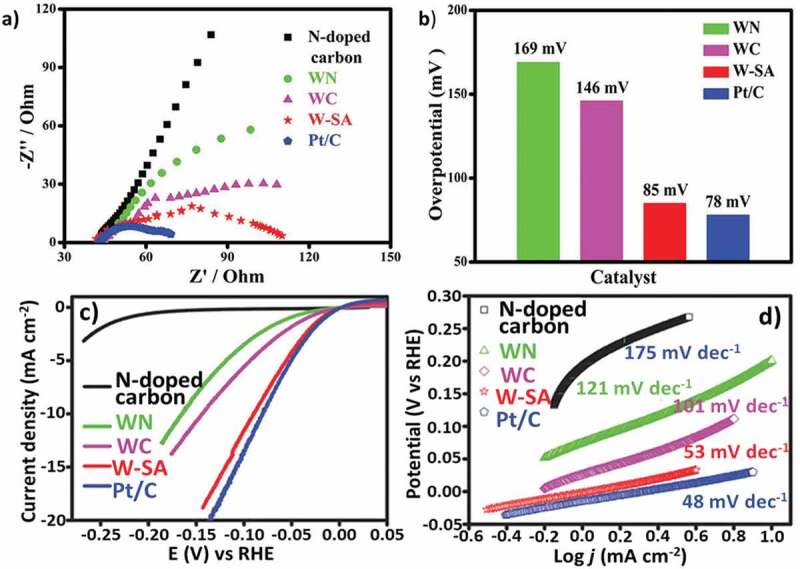
(a) Nyquist plots of W-SAC, (b) Overpotential for W-SAC compared with WC, WN, and 20% Pt/C, (c) LSV curve of the W-SAC for HER performance in alkaline condition (0.1 M KOH), (d) Tafel slope of materials. (Reprinted with permission from Ref [159]. Copyrights 2018) John Wiley and Sons).

To analyze the catalytic mechanism of SAC, Fu et al. [157] experimentally proved that Au_1_ and Pt_1_/CeO_2_ dispersed metal sites are the actual active centers and overturned the well-established fact that nanoparticles are the main active sites. This research nourished further interest in using different SAC [158]. For example, Chen et al. [159] developed a single tungsten atom on MOF-derived N-doped carbon that exhibited excellent HER activity with 85 mV of overpotential @ 10 mA/cm^2^. The Nyquist plot also indicated faster charge transfer capacity of W-SAC during HER process as compared with WC and WN (Figure 14). SACs are ideal catalysts and can enhance atomic efficiency with the maximum utilization of atoms of catalytic metals. In another report, Qiu et al. [160] synthesized single-atom Ni-doped graphene for HER in an acidic medium that showed very low overpotential (50 mV), much better than the Ni-based conventional catalysts. In addition, the catalyst showed improved stability and retained 90% of initial activity after 120 h. This study showed that the electronic and geometric structure of the catalyst where the sp-d orbital charge transfer between the nickel and carbon atom offered better performance. Recently, Ji et al. [161] synthesized highly dispersible single erbium atom catalysts over carbon nitride nanotube surface by using a coordination strategy. XAFS, transmission electron microscopy (TEM) and density functional theory (DFT) calculations proved the significant role of single erbium atom sites towards CO_2_ photoreduction.

Although SACs are fascinating, one of the main issues in these systems is to recognize the active sites and the need for special and expensive characterization methods which still keep the process a state-of-the-art reaction

### 5.4. Carbon nitride (CN_x_) based catalysts

**Figure 15.**
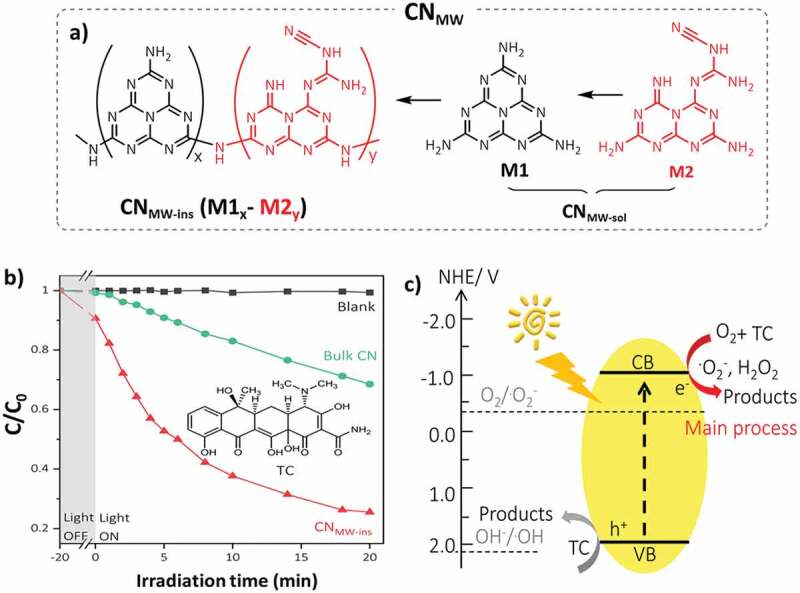
(a) Scheme of CN synthesized by MW process, (b) Absorbance of Tetracycline at 357 nm as a function of time during photocatalytic oxidation reaction using bulk CN and CN_MW-ins_ catalyst, (c) Proposed Mechanism of photocatalytic oxidation of Tetracycline. (Reprinted with permission from Ref [181]. Copyrights 2021, springer nature).

Carbon nitride in its graphitic form is known to be a promising photocatalytic material for CO_2_ reduction and H_2_ generation [162,163]. It exhibits an ideal set of properties such as visible light absorption, tunable band gap, proper redox levels (CB at −1.1 eV and VB at 1.6 eV, NHE), and excellent chemical stability [164]. In addition, the synthesis of carbon nitride is cost-effective and can be obtained simply by thermal polymerization of suitable precursors (urea, melamine, dicyanamide, polyethyleneimine, triazoles, triazines, tetrazoles, etc.) [165,166]. Lin et al. studied different forms of carbon nitride and categorized them as triazine-based crystalline CN, heptazine-based crystalline CN, and CN with both triazine and heptazine units. These three forms along with their synthesis method and applications in photocatalytic water splitting are discussed in detail elsewhere [167]. Dias et al. reported the effects of thermal treatment and subsequent generation of mid-gap states near VB of C_3_N_4_ material on H_2_ evolution. C_3_N_4_ material was prepared and subjected to different post-thermal treatments (@ 620, 650, 680, and 700° C) under an N_2_ atmosphere. The experimental data proved successive enhancement in photocatalytic HER with an increase in temperature except for CN-700. The improvement in performance with an increase in the temperature was due to the trap states produced from N vacancies that enable better charge separation. To know the anomalous behavior of CN-700, diffuse reflectance transient spectroscopic (DR-TS) experiments were carried out on samples CN@650 and CN@700 in the presence and absence of triethanolamine (TEOA). The studies revealed that the holes accumulated in the mid-gap states of the CN@650 sample possess the good potential to scavenge electrons from TEOA and not water, indicating photocatalytic H_2_ evolution and CO_2_ reduction can be controlled by the light-harvesting capacity of the materials and subsequent generation of charge carriers [168]. In order to accomplish this, wide research has been carried out to optimize the C_3_N_4_ stoichiometry. Talapaneni et al. reported mesoporous CN with a large N/C ratio (1.80) by using an aminoguanidine hydrochloride precursor. The authors claimed that the presence of free -NH_2_ groups on the wall structure of the catalyst led to the high catalytic activity for Friedel-Crafts hexanoylation of benzene and also produced a high yield of hexanophenone in less time, mainly due to the presence of free amine groups on the wall structure of the catalyst. The materials outperform the nonporous CN and CN with less N content [169]. However, these materials were not tried for photocatalytic hydrogen production and CO_2_ reduction. Since most of the pure CN syntheses are carried out by using thermal polymerization of various precursors, the intrinsic properties like crystallinity and inherent surface area of bulk CN depend on ramping rate as well. Ni et al. synthesized bulk CN with different ramping rates and observed that with the fast heating rate, the crystallinity and surface area improved. This leads to improved H_2_ generation (121 µmol/g/h) as compared with CN prepared at a slow heating rate (38.7 µmol/g/h) [170]. Various studies on doping CN to achieve effective photocatalytic H_2_ evolution were also reported. For example, Yue et al. doped Zn on C_3_N_4_ and reported 10 times higher yield of hydrogen gas (59.5 µmol/h) compared to undoped C_3_N_4_ (5.5 µmol/h), implying that the zinc present in the C_3_N_4_ matrix captured electrons from the CB of C_3_N_4_ thereby assisting the separation of photogenerated charge carriers and increasing H_2_ production [171]. Other dopants like Na with an evolution rate of 143 μmol h^−1^ (catalyst quantity −20 mg) [172], Fe with 16.2 mmol g^−1^h^−1^ [173], P with 67 μmol h^−1^ (catalyst quantity 100 mg) [174], Co with 1208 μmol g^−1^h^−1^ [175] and bimetals like Ag-Cu with 738 µmol g^−1^h^−1^ [176] were also reported. Although heteroatom or transition or noble metal atom doping of intrinsic C_3_N_4_ stoichiometry is considered a promising strategy for photocatalytic activity, extensive research has been carried out to optimize the physical and chemical properties of C_3_N_4_ material. Mane et al. focused more on developing CN materials with different stoichiometries using various N-rich cyclic precursors, for example, 3-amino-1,2,4-triazole (C_3_N_5_) [177], aminoguanidine hydrochloride (C_3_N_6_) [178], and 5-amino tetrazole [179] for various applications. The authors reported the synthesis of CN with C_3_N_5_ stoichiometry which claimed excellent performance of 801 µmol production of H_2_ gas within 3 h of visible light irradiation. This activity was ascribed to the combination of narrow bandgap which enhances absorption and highly ordered porous structure, resulting in a higher surface area. As discussed, stoichiometry in carbon nitride can majorly alter the electronic properties of this polymer semiconductor. Ma et al. reported a unique C_5_N_2_ stoichiometry with abundant C=N (imine) linkages that offered more delocalized electrons which changed the VB and CB positions [180]. The downshift in CB/VB positions due to C=N linkers eliminated the competitive side reaction of H_2_ generation and resulted in photocatalytic H_2_O_2_ production (1550 µmol/L/h) with 15.4% quantum efficiency, without using any sacrificial agents/co-catalysts. Very recently, Huang et al. [181] reported microwave-assisted synthesis of CN (CN_MW_) and confirmed the coexistence of two well-defined structural motifs: melem (M1) as well as incompletely condensed melem with cyanide termination (M2). Furthermoe, by using ethanol solvent, highly polymerized insoluble product (CN_MW-ins_, yield 70.1%) and partially polymerized soluble product (CN_MW-sol_, yield 12.4%) were synthesized for photocatalytic oxidation of tetracycline. High-end characterization techniques such as quadrupole-time of flight (Q-TOF) mass spectrometry (MS), HPLS, solid state NMR, XPS along with FTIR revealed the dominant contribution of M1 and M2 in light harvesting and charge separation, respectively. Highly polymerized CN_MW-INS_ showed higher photocatalytic activity than bulk CN, and surprisingly, oxygen substrate played a major role in the photo-excited process through the electronic coupling process (Figure 15) [181].

In photocatalytic reactions, co-catalyst addition on the surface of the semiconductor plays a critical role and it is believed that all surface reactions are happening at the interface of the co-catalyst and semiconductor. Bulk CN when loaded with Pt co-catalyst (photo-deposited) could produce H_2_ at a rate of 108 µmol/h/g, under visible light irradiation [182]. Wang et al. increased the rate to 685 µmol/h/g (almost 7 times) by carefully modifying the interface between bulk CN and Pt nanoparticles using a small amount of nitrogen-doped carbon (C-N) transition layer. The authors credit the enhanced photo-activity to the rapid charge transfer of photo-generated electrons from bulk CN to Pt co-catalyst via the (C-N) layer, which reduces the activation barrier [182]. It was not a surprise that very poor (8 µmol/h/g) activity was recorded for bulk CN-(C-N) samples without Pt. Apart from doping methods, self-doping techniques were also reported to improve the electronic band structure and increase visible light absorption [183]. The carbon self-doped g-C_3_N_4_ photocatalyst exhibited better photocatalytic efficiency as compared with nonmetal-doped TiO_2_, BiOBr, (BiO)_2_CO_3_, and even porous g-C_3_N_4_. This exceptional performance was attributed to the enhanced electrical conductivity of g-C_3_N_4_ due to the delocalized π bonds resulting in reduced charge recombination [184]. In g-C_3_N_4_, nitrogen self-doped materials exhibited identical results, yielding 44.28 µmol h^−1^ of hydrogen gas, which is 4.6 times higher than pure g-C_3_N_4_ [185]. Despite its optimal band edge positions, excellent stability, tunable composition, and band gap, further effective strategies need to be developed to reduce the recombination rate and improve the product yield to realize its use in commercial applications.

## 6. Conclusion and future prospects

In summary, this work reviews the adverse impacts of increasing CO_2_ concentration and underlines the importance of CO_2_ capture and its subsequent reduction to value-added products. It presented the fundamentals of CO_2_ conversion and H_2_ generation, critical factors affecting both the reactions, and properties required to realize ideal catalyst materials, and also discussed the performance of various materials towards solar fuel generation. Different possible routes of CO_2_ adsorption on solid catalyst surface have been explained, and various CO_2_ reduction mechanisms are discussed. It is clear that increasing the efficiency of this process is very challenging, kinetically, and the design of nanocatalyst materials with large surface area, proper redox levels, more visible light absorption, multielectron transfer ability, and chemical stability plays a key role. Significant progress has been made in terms of the development of materials by improving the oxidation states, mixing metal ions, reducing particle size, thereby increasing catalytic surface area, and so on. In this work, the performance of catalyst materials, such as TiO_2_, metal chalcogenides, MOFs, single-atom catalysts, and carbon nitrides is reviewed. Multielectron and proton transfer from the catalyst surface to adsorbed CO_2_ molecule is a rate-determining step in photocatalytic CO_2_ reduction. Therefore, proper design of the catalyst surface to increase the feasibility of CO_2_ adsorption and thereby lower the kinetic barrier for multielectron transfer can be a good strategy for improving the efficiency of this process. The scientific community is paying specific attention to the structurally and electronically modified catalyst design to achieve maximum efficiency. Some emerging novel fields such as nanobiotechnology in which high surface area nanoparticles are integrated with microalgae can play a crucial role. In this system, a high quantity of CO_2_ biofixation can be achieved by microalgae and high surface area NPs increases CO_2_ adsorption capacity in liquid medium. However, it should be emphasized that though enormous advances have been achieved, there is still a long way to get these materials to be applied for the bulk-scale application. The major problem is the high cost of techniques used in the synthesis of these catalyst materials, which often result in the formation of a limited amount of material, thus limiting their commercial production and uses. Although doped TiO_2_ and non-TiO_2_-based visible light absorbing materials have been developed, there is an urgent need to invent new materials that can harvest more visible photons, match perfect redox potentials, and show high stability. Apart from the materials, methods, and drawbacks discussed in this article, many techniques like in situ Fourier-transform infrared spectroscopy, XAFS, and in-situ EPR can better characterise the complex reaction mechanism of CO_2_ conversion to solar fuels.

## Abbreviations

**Abbreviation**: **Meaning**
HER: Hydrogen Evolution Reaction
CC: Carbon Dioxide Capture
GHGs: Green House Gases
EPR: Electron Paramagnetic Resonance
QDs: Quantum Dots
NF’s: Nano Fibers
NP’s: Nanoparticles
SAC: Single Atom Catalyst
THSs: TiO_2_ Hierarchical Spheres
CASTEP: Cambridge Serial Total Energy Package (software)
IL’s: Ionic Liquids
RGO: Reduced Graphene Oxide
MOF’s: Metal Organic Frameworks
CN: Carbon Nitride
DR-TS: Diffuse Reflectance Transient spectroscopic
TEOA: Triethanolamine

## References

[1] Gupta A, Paul A. Carbon capture and sequestration potential in India: a comprehensive review. Energy Procedia. 2019;160:848–855.

[2] Kumar P, Laishram D, Sharma RK, et al. Boosting photocatalytic activity using carbon nitride based 2D/2D van der Waals heterojunctions. Chem Mater. 2021;33(23):9012–9092. DOI:10.1021/acs.chemmater.1c03166

[3] Draper AM, Weissburg MJ. Impacts of global warming and elevated CO2 on sensory behavior in predator-prey interactions: a review and synthesis. Front Ecol Evol. 2019;7:72.

[4] Singh G, Kim IY, Lakhi KS, et al. Heteroatom functionalized activated porous biocarbons and their excellent performance for CO2 capture at high pressure. J Mater Chem A. 2017;5(40):21196–21204. DOI:10.1039/C7TA07186H

[5] Pachauri R, Meyer L. Climate change 2014: synthesis report. Contribution of working groups I, II and III to the fifth assessment report of the intergovernmental panel on climate change. 2014.

[6] Peter SC. Reduction of CO_2_ to chemicals and fuels: a solution to global warming and energy crisis. ACS Energy Lett. 2018;3(7):1557–1561.

[7] Lakhi K, Cha W, Joseph S, et al. Cage type mesoporous carbon nitride with large mesopores for CO2 capture. CatalToday. 2015;243:209–217.

[8] Singh G, Lee J, Bahadur R, et al. Highly graphitized porous biocarbon nanosheets with tunable micro-meso interfaces and enhanced layer spacing for CO_2_ capture and LIBs. Chem Eng J. 2022;433:134464.

[9] Ramadass K, Sathish CI, Singh G, et al. Morphologically tunable nanoarchitectonics of mixed kaolin-halloysite derived nitrogen-doped activated nanoporous carbons for supercapacitor and CO_2_ capture applications. Carbon. 2022;192:133–144.

[10] Sathish C, Kothandam G, Selvarajan P, et al. Ordered mesoporous boron carbon nitrides with tunable mesopore nanoarchitectonics for energy storage and CO_2_ adsorption properties. Adv Sci. 2022;9(16):2105603. DOI:10.1002/advs.202105603 PMC9165510 35384377

[11] Ismail IS, Singh G, Smith P, et al. Oxygen functionalized porous activated biocarbons with high surface area derived from grape marc for enhanced capture of CO_2_ at elevated-pressure. Carbon. 2020;160:113–124.

[12] Singh G, Bahadur R, Ruban AM, et al. Synthesis of functionalized nanoporous biocarbons with high surface area for CO_2_ capture and supercapacitor applications. Green Chem. 2021;23(15):5571–5583. DOI:10.1039/D1GC01376A

[13] Singh G, Maria Ruban A, Geng X, et al. Recognizing the potential of K-salts, apart from KOH, for generating porous carbons using chemical activation. Chem Eng J. 2023;451:139045.

[14] Kanniche M, Gros-Bonnivard R, Jaud P, et al. Pre-combustion, post-combustion and oxy-combustion in thermal power plant for CO_2_ capture. Appl Therm Eng. 2010;30(1):53–62. DOI:10.1016/j.applthermaleng.2009.05.005

[15] Singh G, Lee J, Karakoti A, et al. Emerging trends in porous materials for CO_2_ capture and conversion. Chemical Society Rev. 2020;49(13):4360–4404. 10.1039/d0cs00075b 32458938

[16] Su T, Shao Q, Qin Z, et al. Role of interfaces in two-dimensional photocatalyst for water splitting. ACS Catal. 2018;8(3):2253–2276. DOI:10.1021/acscatal.7b03437

[17] Prasad C, Tang H, Liu QQ, et al. An overview of semiconductors/layered double hydroxides composites: properties, synthesis, photocatalytic and photoelectrochemical applications. J Mol Liq. 2019;289:111114.

[18] Singh AK, Montoya JH, Gregoire JM, et al. Robust and synthesizable photocatalysts for CO2 reduction: a data-driven materials discovery. Nat Commun. 2019;10(1):1–9. DOI:10.1038/s41467-019-08356-1 30683857 PMC6347635

[19] Li X, Yu J, Jaroniec M. Hierarchical photocatalysts. Chem Soc Rev. 2016;45(9):2603–2636. 26963902 10.1039/c5cs00838g

[20] Kayfeci M, Keçebaş A, Bayat M. Solar hydrogen. Elsevier; 2019. Chapter 3, Hydrogen production; p. 45–83.

[21] Gujral HS, Singh G, Baskar AV, et al. Metal nitride-based nanostructures for electrochemical and photocatalytic hydrogen production. Sci Technol Adv Mater. 2022;23(1):76–119. DOI:10.1080/14686996.2022.2029686 35309252 PMC8928826

[22] Jenck JF, Agterberg F, Droescher MJ. Products and processes for a sustainable chemical industry: a review of achievements and prospects. Green Chem. 2004;6(11):544–556.

[23] Hydrogen Industry application: past, present and future. Available from: https://wha-international.com/hydrogen-in-industry/

[24] International Energy Agency Report. The future of hydrogen 2019. Available from: https://www.iea.org/fuels-and-technologies/hydrogen

[25] Basheer AA, Ali I. Water photo splitting for green hydrogen energy by green nanoparticles. Int J Hydrogen Energy. 2019;44(23):11564–11573.

[26] Kamat PV. Manipulation of charge transfer across semiconductor interface. A criterion that cannot be ignored in photocatalyst design. J Phys Chem Lett. 2012;3(5):663–672. 26286163 10.1021/jz201629p

[27] Ganguly P, Harb M, Cao Z, et al. 2D nanomaterials for photocatalytic hydrogen production. ACS Energy Lett. 2019;4(7):1687–1709. DOI:10.1021/acsenergylett.9b00940

[28] Wang X, Wang F, Sang Y, et al. Full‐spectrum solar‐light‐activated photocatalysts for light–chemical energy conversion. Adv Energy Mater. 2017;7(23):1700473. DOI:10.1002/aenm.201700473

[29] LeValley TL, Richard AR, Fan M. The progress in water gas shift and steam reforming hydrogen production technologies – a review. Int J Hydrogen Energy. 2014;39(30):16983–17000.

[30] Sengodan S, Lan R, Humphreys J, et al. Advances in reforming and partial oxidation of hydrocarbons for hydrogen production and fuel cell applications. Renew Sust Energ Rev. 2018;82:761–780.

[31] Rabenstein G, Hacker V. Hydrogen for fuel cells from ethanol by steam-reforming, partial-oxidation and combined auto-thermal reforming: a thermodynamic analysis. J Power Sources. 2008;185(2):1293–1304.

[32] Chaubey R, Sahu S, James OO, et al. A review on development of industrial processes and emerging techniques for production of hydrogen from renewable and sustainable sources. Renew Sust Energ Rev. 2013;23:443–462.

[33] Łukajtis R, Hołowacz I, Kucharska K, et al. Hydrogen production from biomass using dark fermentation. Renew Sust Energ Rev. 2018;91:665–694.

[34] Akhlaghi N, Najafpour-Darzi G. A comprehensive review on biological hydrogen production. Int J Hydrogen Energy. 2020;45(43):22492–22512.

[35] Cao L, Iris K, Xiong X, et al. Biorenewable hydrogen production through biomass gasification: a review and future prospects. Environ Res. 2020;186:109547. 32335432 10.1016/j.envres.2020.109547

[36] Nikolaidis P, Poullikkas A. A comparative overview of hydrogen production processes. Renew Sust Energ Rev. 2017;67:597–611.

[37] Hosseini SE, Wahid MA. Hydrogen from solar energy, a clean energy carrier from a sustainable source of energy. Int J Energy Res. 2020;44(6):4110–4131.

[38] Burton N, Padilla R, Rose A, et al. Increasing the efficiency of hydrogen production from solar powered water electrolysis. Renew Sust Energ Rev. 2021;135:110255.

[39] Rönsch S, Schneider J, Matthischke S, et al. Review on methanation – from fundamentals to current projects. Fuel. 2016;166:276–296.

[40] Muscatello ESM AC. Mars in situ resource utilization technology evaluation. 50th AIAA Aerospace Sciences Meeting including the New Horizons Forum and Aerospace Exposition; 2012, Nashville, TN.

[41] Wagner A, Sahm CD, Reisner E. Towards molecular understanding of local chemical environment effects in electro-and photocatalytic CO_2_ reduction. Nat Catal. 2020;3(10):775–786.

[42] Burkart MD, Hazari N, Tway CL, et al. Opportunities and challenges for catalysis in carbon dioxide utilization. ACS Catal. 2019;9(9):7937–7956. DOI:10.1021/acscatal.9b02113

[43] Fu J, Wang S, Wang Z, et al. Graphitic carbon nitride based single-atom photocatalysts. Front Phys. 2020;15(3):1–14. DOI:10.1007/s11467-019-0950-z

[44] Ran J, Jaroniec M, Qiao SZ. Cocatalysts in semiconductor‐based photocatalytic CO_2_ reduction: achievements, challenges, and opportunities. Adv Mater. 2018;30(7):1704649. 10.1002/adma.201704649 29315885

[45] Bahadori E, Tripodi A, Villa A, et al. High pressure photoreduction of CO_2_: effect of catalyst formulation, hole scavenger addition and operating conditions. Catalysts. 2018;8(10):430. DOI:10.3390/catal8100430

[46] Jiao X, Zheng K, Hu Z, et al. Broad-spectral-response photocatalysts for CO_2_ reduction. ACS Cent Sci. 2020;6(5):653–660. DOI:10.1021/acscentsci.0c00325 32490183 PMC7256938

[47] Zhou R, Guzman MI. CO_2_ reduction under periodic illumination of ZnS. J Phys Chem C. 2014;118(22):11649–11656.

[48] Ogata T, Yanagida S, Brunschwig BS, et al. Mechanistic and kinetic studies of cobalt macrocycles in a photochemical CO2 reduction system: evidence of Co-CO_2_ adducts as intermediates. J Am Chem Soc. 1995;117(25):6708–6716. DOI:10.1021/ja00130a009

[49] Mishra A, Basu S, Shetti NP, et al. Nanoscale Materials in Water Purification. Elsevier; 2019.Chapter 37, Photocatalysis of graphene and carbon nitride-based functional carbon quantum dots; p. 759–781.

[50] Hao X, Jin Z, Xu J, et al. Functionalization of TiO_2_ with graphene quantum dots for efficient photocatalytic hydrogen evolution. Superlattices Microstruct. 2016;94:237–244.

[51] Gliniak J, Lin JH, Chen YT, et al. Sulfur‐doped graphene oxide quantum dots as photocatalysts for hydrogen generation in the aqueous phase. ChemSuschem. 2017;10(16):3260–3267. DOI:10.1002/cssc.201700910 28656618

[52] Sorcar S, Hwang Y, Grimes CA, et al. Highly enhanced and stable activity of defect-induced titania nanoparticles for solar light-driven CO_2_ reduction into CH4. Mater Today. 2017;20(9):507–515. DOI:10.1016/j.mattod.2017.09.005

[53] Saw K, Aznan N, Yam F, et al. New insights on the Burstein-Moss shift and band gap narrowing in indium-doped zinc oxide thin films. PLoS One. 2015;10(10):e0141180. DOI:10.1371/journal.pone.0141180 26517364 PMC4627753

[54] Shen Z, Xia Q, Li Y, et al. Adsorption-enhanced nitrogen-doped mesoporous CeO2 as an efficient visible-light-driven catalyst for CO_2_ photoreduction. J CO2 Util. 2020;39:101176.

[55] Sayed M, Xu F, Kuang P, et al. Sustained CO_2_-photoreduction activity and high selectivity over Mn, C-codoped ZnO core-triple shell hollow spheres. Nat Commun. 2021;12(1):1–10. DOI:10.1038/s41467-021-25007-6 34400631 PMC8368040

[56] Nematollahi R, Ghotbi C, Khorasheh F, et al. Ni-Bi co-doped TiO_2_ as highly visible light response nano-photocatalyst for CO_2_ photo-reduction in a batch photo-reactor. J CO2 Util. 2020;41:101289.

[57] Moradi M, Khorasheh F, Larimi A. Pt nanoparticles decorated Bi-doped TiO_2_ as an efficient photocatalyst for CO2 photo-reduction into CH4. Solar Energy. 2020;211:100–110.

[58] Fu J, Liu K, Jiang K, et al. Graphitic carbon nitride with dopant induced charge localization for enhanced photoreduction of CO_2_ to CH_4_. Adv Sci. 2019;6(18):1900796. DOI:10.1002/advs.201900796 PMC6755511 31559128

[59] Li R, Zhang W, Zhou K. Metal–organic‐framework‐based catalysts for photoreduction of CO_2_. Adv Mater. 2018;30(35):1705512. 10.1002/adma.201705512 29894012

[60] Lais A, Gondal M, Dastageer M, et al. Experimental parameters affecting the photocatalytic reduction performance of CO_2_ to methanol: a review. Int J Energy Res. 2018;42(6):2031–2049. DOI:10.1002/er.3965

[61] She X, Liu L, Ji H, et al. Template-free synthesis of 2D porous ultrathin nonmetal-doped g-C_3_N_4_ nanosheets with highly efficient photocatalytic H_2_ evolution from water under visible light. Appl Catal B Environ. 2016;187:144–153.

[62] Fu J, Jiang K, Qiu X, et al. Product selectivity of photocatalytic CO_2_ reduction reactions. Mater Today. 2020;32:222–243.

[63] Tahir M, Amin NS. Recycling of carbon dioxide to renewable fuels by photocatalysis: prospects and challenges. Renew Sust Energ Rev. 2013;25:560–579.

[64] Omadoko O, Scott D, Hickman R, et al. Simple photoreduction of carbon dioxide to formic acid and true quantum yield. Phys Chem Chem Phys. 2020;22(8):4632–4639. DOI:10.1039/C9CP06707H 32052000

[65] Neaţu Ş, Maciá-Agulló JA, Garcia H. Solar light photocatalytic CO_2_ reduction: general considerations and selected bench-mark photocatalysts. Int J Mol Sci. 2014;15(4):5246–5262. 24670477 10.3390/ijms15045246 PMC4013561

[66] Gonell F, Puga AV, Julian-Lopez B, et al. Copper-doped titania photocatalysts for simultaneous reduction of CO_2_ and production of H_2_ from aqueous sulfide. Appl Catal B Environ. 2016;180:263–270.

[67] Zhang Q, Lin C-F, Jing YH, et al. Photocatalytic reduction of carbon dioxide to methanol and formic acid by graphene-TiO_2_. J Air Waste Manage Assoc. 2014;64(5):578–585. DOI:10.1080/10962247.2013.875958 24941706

[68] Monteiro MC, Philips MF, Schouten KJP, et al. Efficiency and selectivity of CO_2_ reduction to CO on gold gas diffusion electrodes in acidic media. Nat Commun. 2021;12(1):1–7. DOI:10.1038/s41467-021-24936-6 34400626 PMC8368099

[69] Bhattacharyya K, Mane GP, Rane V, et al. Selective CO_2_ photoreduction with Cu-doped TiO_2_ photocatalyst: delineating the crucial role of Cu-oxidation state and oxygen vacancies. J Phys Chem C. 2021;125(3):1793–1810. DOI:10.1021/acs.jpcc.0c08441

[70] Thompson W, Fernandez ES, Maroto-Valer M. Probability Langmuir-Hinshelwood based CO_2_ photoreduction kinetic models. Chem Eng J. 2020;384:123356.

[71] Hossain MM, Raupp GB. Polychromatic radiation field model for a honeycomb monolith photocatalytic reactor. Chem Eng Sci. 1999;54(15–16):3027–3034.

[72] Wang Z, Liu J, Dai Y, et al. CFD modeling of a UV-LED photocatalytic odor abatement process in a continuous reactor. J Hazard Mater. 2012;215:25–31. 22417398 10.1016/j.jhazmat.2012.02.021

[73] Liu S, Zhao Z, Wang Z. Photocatalytic reduction of carbon dioxide using sol–gel derived titania-supported CoPc catalysts. Photochem Photobiol Sci. 2007;6(6):695–700. 17549273 10.1039/b613098d

[74] Gattrell M, Gupta N, Co A. A review of the aqueous electrochemical reduction of CO_2_ to hydrocarbons at copper. J Electroanal Chem. 2006;594(1):1–19.

[75] Habisreutinger SN, Schmidt‐mende L, Stolarczyk JK. Photocatalytic reduction of CO_2_ on TiO_2_ and other semiconductors. Angewandte Chemie. 2013;52(29):7372–7408. 23765842 10.1002/anie.201207199

[76] Wang L, Chen W, Zhang D, et al. Surface strategies for catalytic CO_2_ reduction: from two-dimensional materials to nanoclusters to single atoms. Chem Soc Rev. 2019;48(21):5310–5349. DOI:10.1039/C9CS00163H 31588933

[77] Chang X, Wang T, Gong J. CO_2_ photo-reduction: insights into CO_2_ activation and reaction on surfaces of photocatalysts. Energy Environ Sci. 2016;9(7):2177–2196.

[78] Freund H-J, Roberts MW. Surface chemistry of carbon dioxide. Surf Sci Rep. 1996;25(8):225–273.

[79] Mori K, Yamashita H, Anpo M. Photocatalytic reduction of CO_2_ with H_2_O on various titanium oxide photocatalysts. RSC Adv. 2012;2(8):3165–3172.

[80] Shkrob IA, Marin TW, He H, et al. Photoredox reactions and the catalytic cycle for carbon dioxide fixation and methanogenesis on metal oxides. J Phys Chem C. 2012;116(17):9450–9460. DOI:10.1021/jp300122v

[81] Pougin A, Dilla M, Strunk J. Identification and exclusion of intermediates of photocatalytic CO_2_ reduction on TiO_2_ under conditions of highest purity. Phys Chem Chem Phys. 2016;18(16):10809–10817. 26971862 10.1039/c5cp07148h

[82] Civiš S, Knížek A, Ivanek O, et al. The origin of methane and biomolecules from a CO_2_ cycle on terrestrial planets. Nat Astron. 2017;1(10):721–726. DOI:10.1038/s41550-017-0260-8

[83] Ji Y, Luo Y. New mechanism for photocatalytic reduction of CO_2_ on the anatase TiO_2_ (101) surface: the essential role of oxygen vacancy. J Am Chem Soc. 2016;138(49):15896–15902. 27960337 10.1021/jacs.6b05695

[84] Lee CH, Lee SU. Electrocatalysts for fuel cells and hydrogen evolution-theory to design. London: IntechOpen; 2018. Theoretical basis of electrocatalysis; p. 13.

[85] Li C, Baek J-B. Recent advances in noble metal (Pt, Ru, and Ir)-based electrocatalysts for efficient hydrogen evolution reaction. ACS Omega. 2019;5(1):31–40. 31956748 10.1021/acsomega.9b03550 PMC6963895

[86] Jiang L, Wang Y, Feng C. Application of photocatalytic technology in environmental safety. Procedia Eng. 2012;45:993–997.

[87] Ohno T, Sarukawa K, Tokieda K, et al. Morphology of a TiO_2_ photocatalyst (Degussa, P-25) consisting of anatase and rutile crystalline phases. J Catal. 2001;203(1):82–86. DOI:10.1006/jcat.2001.3316

[88] Jing L, Li S, Song S, et al. Investigation on the electron transfer between anatase and rutile in nano-sized TiO_2_ by means of surface photovoltage technique and its effects on the photocatalytic activity. Sol Energy Mater Sol Cells. 2008;92(9):1030–1036. DOI:10.1016/j.solmat.2008.03.003

[89] Ikram M, Hassan J, Raza A, et al. Photocatalytic and bactericidal properties and molecular docking analysis of TiO_2_ nanoparticles conjugated with Zr for environmental remediation. RSC Adv. 2020;10(50):30007–30024. DOI:10.1039/D0RA05862A 35518250 PMC9056309

[90] Dette C, Pérez-Osorio MA, Kley CS, et al. TiO_2_ anatase with a bandgap in the visible region. Nano Lett. 2014;14(11):6533–6538. DOI:10.1021/nl503131s 25252265

[91] Dong H, Zeng G, Tang L, et al. An overview on limitations of TiO_2_-based particles for photocatalytic degradation of organic pollutants and the corresponding countermeasures. Water Res. 2015;79:128–146. 25980914 10.1016/j.watres.2015.04.038

[92] Bhattacharyya A, Kawi S, Ray M. Photocatalytic degradation of orange-II by TiO_2_ catalysts supported on adsorbents. CatalToday. 2004;98(3):431–439.

[93] Maeda K, Lu D, Domen K. Direct water splitting into hydrogen and oxygen under visible light by using modified TaON photocatalysts with d^0^ electronic configuration. Chem–A Eur J. 2013;19(16):4986–4991. 10.1002/chem.201300158 23553815

[94] Sarkar A, Gracia-Espino E, Wågberg T, et al. Photocatalytic reduction of CO_2_ with H_2_O over modified TiO_2_ nanofibers: understanding the reduction pathway. Nano Res. 2016;9(7):1956–1968. DOI:10.1007/s12274-016-1087-9

[95] Ikeue K, Yamashita H, Anpo M, et al. Photocatalytic reduction of CO_2_ with H_2_O on Ti− β zeolite photocatalysts: effect of the hydrophobic and hydrophilic properties. J Phys Chem B. 2001;105(35):8350–8355. DOI:10.1021/jp010885g

[96] Anpo M. Photocatalytic reduction of CO_2_ with H_2_O on highly dispersed Ti-oxide catalysts as a model of artificial photosynthesis. J CO2 Util. 2013;1:8–17.

[97] Tseng I-H, Chang W-C, Wu JC. Photoreduction of CO_2_ using sol–gel derived titania and titania-supported copper catalysts. Appl Catal B Environ. 2002;37(1):37–48.

[98] Li X, Zhuang Z, Li W, et al. Photocatalytic reduction of CO_2_ over noble metal-loaded and nitrogen-doped mesoporous TiO_2_. Appl Catal A Gen. 2012;429:31–38.

[99] Michaelson HB. The work function of the elements and its periodicity. J Appl Phys. 1977;48(11):4729–4733.

[100] Low J, Cheng B, Yu J. Surface modification and enhanced photocatalytic CO_2_ reduction performance of TiO_2_: a review. Appl Surface Sci. 2017;392:658–686.

[101] Xie S, Wang Y, Zhang Q, et al. MgO-and Pt-promoted TiO_2_ as an efficient photocatalyst for the preferential reduction of carbon dioxide in the presence of water. ACS Catal. 2014;4(10):3644–3653. DOI:10.1021/cs500648p

[102] Li K, An X, Park KH, et al. A critical review of CO_2_ photoconversion: catalysts and reactors. CatalToday. 2014;224:3–12.

[103] Li Y, Wang W-N, Zhan Z, et al. Photocatalytic reduction of CO_2_ with H_2_O on mesoporous silica supported Cu/TiO2 catalysts. Appl Catal B Environ. 2010;100(1–2):386–392. DOI:10.1016/j.apcatb.2010.08.015

[104] Tseng I-H, Wu JC, Chou H-Y. Effects of sol–gel procedures on the photocatalysis of Cu/TiO_2_ in CO_2_ photoreduction. J Catal. 2004;221(2):432–440.

[105] Roy SC, Varghese OK, Paulose M, et al. Toward solar fuels: photocatalytic conversion of carbon dioxide to hydrocarbons. ACS Nano. 2010;4(3):1259–1278. DOI:10.1021/nn9015423 20141175

[106] Liu Y, Zhou S, Li J, et al. Photocatalytic reduction of CO_2_ with water vapor on surface La-modified TiO_2_ nanoparticles with enhanced CH4 selectivity. Appl Catal B Environ. 2015;168:125–131.

[107] Gao L, Li Y, Ren J, et al. Passivation of defect states in anatase TiO_2_ hollow spheres with Mg doping: realizing efficient photocatalytic overall water splitting. Appl Catal B Environ. 2017;202:127–133.

[108] Jakob M, Levanon H, Kamat PV. Charge distribution between UV-irradiated TiO_2_ and gold nanoparticles: determination of shift in the Fermi level. Nano Lett. 2003;3(3):353–358.

[109] Ortiz AL, Zaragoza MM, Gutiérrez JS, et al. Silver oxidation state effect on the photocatalytic properties of Ag doped TiO_2_ for hydrogen production under visible light. Int J Hydrogen Energy. 2015;40(48):17308–17315. DOI:10.1016/j.ijhydene.2015.09.058

[110] Yan B, Liu D, Feng X, et al. Ru species supported on MOF‐derived N‐doped TiO_2_/C hybrids as efficient electrocatalytic/photocatalytic hydrogen evolution reaction catalysts. Adv Funct Mater. 2020;30(31):2003007. DOI:10.1002/adfm.202003007

[111] Wang J, Shen Y, Liu S, et al. Single 2D MXene precursor-derived TiO_2_ nanosheets with a uniform decoration of amorphous carbon for enhancing photocatalytic water splitting. Appl Catal B Environ. 2020;270(5):118885.

[112] Hu H, Qian D, Lin P, et al. Oxygen vacancies mediated in-situ growth of noble-metal (Ag, Au, Pt) nanoparticles on 3D TiO_2_ hierarchical spheres for efficient photocatalytic hydrogen evolution from water splitting. Int J Hydrogen Energy. 2020;45(1):629–639. DOI:10.1016/j.ijhydene.2019.10.231

[113] M-C W, P-Y W, Lin T-H, et al. Photocatalytic performance of Cu-doped TiO_2_ nanofibers treated by the hydrothermal synthesis and air-thermal treatment. Appl Surface Sci. 2018;430:390–398.

[114] Mor GK, Varghese OK, Wilke RH, et al. P-Type Cu− Ti− O nanotube arrays and their use in self-biased heterojunction photoelectrochemical diodes for hydrogen generation. Nano Lett. 2008;8(7):1906–1911. DOI:10.1021/nl080572y 18540655

[115] Wu J, Huang Y, Ye W, et al. CO_2_ reduction: from the electrochemical to photochemical approach. Adv Sci. 2017;4(11):1700194. DOI:10.1002/advs.201700194 PMC5700640 29201614

[116] Luo X, Guo Y, Ding F, et al. Significant improvements in CO_2_ capture by pyridine‐containing anion‐functionalized ionic liquids through multiple‐site cooperative interactions. Angew Chem. 2014;126(27):7173–7177. DOI:10.1002/ange.201400957 24899207

[117] Liu Q, Zhou Y, Kou J, et al. High-yield synthesis of ultralong and ultrathin Zn_2_GeO_4_ nanoribbons toward improved photocatalytic reduction of CO_2_ into renewable hydrocarbon fuel. J Am Chem Soc. 2010;132(41):14385–14387. DOI:10.1021/ja1068596 20866065

[118] Yu J, Jin J, Cheng B, et al. A noble metal-free reduced graphene oxide–CDs nanorod composite for the enhanced visible-light photocatalytic reduction of CO_2_ to solar fuel. J Mater Chem A. 2014;2(10):3407–3416. DOI:10.1039/c3ta14493c

[119] An X, Li K, Tang J. Cu2o/Reduced graphene oxide composites for the photocatalytic conversion of CO_2_. ChemSuschem. 2014;7(4):1086–1093. 24574039 10.1002/cssc.201301194 PMC4204277

[120] Ouyang C, Wang X, Wang C, et al. Hierarchically porous Ni_3_S_2_ nanorod array foam as highly efficient electrocatalyst for hydrogen evolution reaction and oxygen evolution reaction. Electrochimica Acta. 2015;174:297–301.

[121] Chung DY, Han JW, Lim D-H, et al. Structure dependent active sites of Ni-xS-y as electrocatalysts for hydrogen evolution reaction. Nanoscale. 2015;7(12):5157–5163. DOI:10.1039/C4NR07648F 25671375

[122] Kukunuri S, Krishnan MR, Sampath S. The effect of structural dimensionality on the electrocatalytic properties of the nickel selenide phase. Phys Chem Chem Phys. 2015;17(36):23448–23459. 26291172 10.1039/c5cp03900b

[123] Tang C, Xie L, Sun X, et al. Highly efficient electrochemical hydrogen evolution based on nickel diselenide nanowall film. Nanotechnology. 2016;27(20):20LT02. DOI:10.1088/0957-4484/27/20/20LT02 27070104

[124] Bhat KS, Nagaraja H. Recent trends and insights in nickel chalcogenide nanostructures for water-splitting reactions. Mater Res Innovations. 2021;25(1):29–52.

[125] Chia X, Sofer Z, Luxa J, et al. Unconventionally layered CoTe_2_ and NiTe_2_ as electrocatalysts for hydrogen evolution. Chem–A Eur J. 2017;23(48):11719–11726. DOI:10.1002/chem.201702753 28791768

[126] Ge Y, Gao S-P, Dong P, et al. Insight into the hydrogen evolution reaction of nickel dichalcogenide nanosheets: activities related to non-metal ligands. Nanoscale. 2017;9(17):5538–5544. DOI:10.1039/C6NR09977G 28405648

[127] Dong G, Zhang L. Porous structure dependent photoreactivity of graphitic carbon nitride under visible light. J Mater Chem. 2012;22(3):1160–1166.

[128] Ovcharov M, Shcherban N, Filonenko S, et al. Hard template synthesis of porous carbon nitride materials with improved efficiency for photocatalytic CO_2_ utilization. Mater Sci Eng B. 2015;202:1–7.

[129] Wang Z, Jiao X, Chen D, et al. Porous Copper/Zinc bimetallic oxides derived from MOFs for efficient photocatalytic reduction of CO_2_ to methanol. Catalysts. 2020;10(10):1127. DOI:10.3390/catal10101127

[130] Ong W-J, Tan L-L, Chai S-P, et al. Graphene oxide as a structure-directing agent for the two-dimensional interface engineering of sandwich-like graphene–g C_3_N_4_ hybrid nanostructures with enhanced visible-light photoreduction of CO_2_ to methane. Chem Comm. 2015;51(5):858–861. DOI:10.1039/C4CC08996K 25429376

[131] Shcherban ND, Filonenko SM, Ovcharov ML, et al. Simple method for preparing of sulfur–doped graphitic carbon nitride with superior activity in CO_2_ photoreduction. ChemistrySelect. 2016;1(15):4987–4993. DOI:10.1002/slct.201601283

[132] Shen M, Zhang L, Wang M, et al. Carbon-vacancy modified graphitic carbon nitride: enhanced CO_2_ photocatalytic reduction performance and mechanism probing. J Mater Chem A. 2019;7(4):1556–1563. DOI:10.1039/C8TA09302D

[133] Zhu X, Ji H, Yi J, et al. A specifically exposed cobalt oxide/carbon nitride 2D heterostructure for carbon dioxide photoreduction. Ind Eng Chem Res. 2018;57(51):17394–17400. DOI:10.1021/acs.iecr.8b04123

[134] Zeng S, Kar P, Thakur UK, et al. A review on photocatalytic CO_2_ reduction using perovskite oxide nanomaterials. Nanotechnology. 2018;29(5):052001. DOI:10.1088/1361-6528/aa9fb1 29214981

[135] Guo Q, Fu L, Yan T, et al. Improved photocatalytic activity of porous ZnO nanosheets by thermal deposition graphene-like g-C_3_N_4_ for CO_2_ reduction with H_2_O vapor. Appl Surface Sci. 2020;509:144773.

[136] Wang S, Zhan J, Chen K, et al. Potassium-doped g-C_3_N_4_ achieving efficient visible-light-driven CO_2_ reduction. ACS Sustainable Chem Eng. 2020;8(22):8214–8222. DOI:10.1021/acssuschemeng.0c01151

[137] Zhang H, Tang Y, Liu Z, et al. Study on optical properties of alkali metal doped g-C_3_N_4_ and their photocatalytic activity for reduction of CO_2_. Chem Phys Lett. 2020;751:137467.

[138] Shi G, Yang L, Liu Z, et al. Photocatalytic reduction of CO_2_ to CO over copper decorated g-C_3_N_4_ nanosheets with enhanced yield and selectivity. Appl Surface Sci. 2018;427:1165–1173.

[139] Tonda S, Kumar S, Kandula S, et al. Fe-doped and-mediated graphitic carbon nitride nanosheets for enhanced photocatalytic performance under natural sunlight. J Mater Chem A. 2014;2(19):6772–6780. DOI:10.1039/c3ta15358d

[140] Qamar MA, Shahid S, Javed M, et al. Highly efficient g-C_3_N_4_/Cr-ZnO nanocomposites with superior photocatalytic and antibacterial activity. J Photochem Photobiol A. 2020;401:112776.

[141] Ong W-J, Tan L-L, Chai S-P, et al. Heterojunction engineering of graphitic carbon nitride (gC_3_N_4_) via Pt loading with improved daylight-induced photocatalytic reduction of carbon dioxide to methane. Dalton Trans. 2015;44(3):1249–1257. DOI:10.1039/C4DT02940B 25415620

[142] Li S, Yan S, Xia Y, et al. Oxidative reactivity enhancement for soot combustion catalysts by co-doping silver and manganese in ceria. Appl Catal A Gen. 2019;570:299–307.

[143] Sun X, Li M, Ren S, et al. Zeolitic imidazolate framework-cellulose nanofiber hybrid membrane as Li-Ion battery separator: basic membrane property and battery performance. J Power Sources. 2020;454:227878.

[144] Cao S, Li Y, Zhu B, et al. Facet effect of Pd cocatalyst on photocatalytic CO_2_ reduction over g-C3N4. J Catal. 2017;349:208–217.

[145] Karuppasamy K, Jothi VR, Vikraman D, et al. Metal-organic framework derived NiMo polyhedron as an efficient hydrogen evolution reaction electrocatalyst. Appl Surface Sci. 2019;478:916–923.

[146] Peters AW, Li Z, Farha OK, et al. Toward inexpensive photocatalytic hydrogen evolution: a nickel sulfide catalyst supported on a high-stability metal–organic framework. ACS Appl Mater Interfaces. 2016;8(32):20675–20681. DOI:10.1021/acsami.6b04729 27487409

[147] Do HH, Van Le Q, Tekalgne MA, et al. Metal–organic framework-derived MoSx composites as efficient electrocatalysts for hydrogen evolution reaction. J Alloys Compd. 2021;852:156952.

[148] Li Y-W, Wu Q, Ma R-C, et al. A Co-MOF-derived Co_9_S_8_@ NS-C electrocatalyst for efficient hydrogen evolution reaction. RSC Adv. 2021;11(11):5947–5957. DOI:10.1039/D0RA10864B 35423155 PMC8694845

[149] Verma P, Stewart JD, Raja R. Recent advances in photocatalytic CO_2_ utilisation over multifunctional metal–organic frameworks. Catalysts. 2020;10(10):1176.

[150] Yang X-F, Qiao B, Li J, et al. Acc Chem Res. 2013;46:1740–1748. DOI:10.1021/ar300361m 23815772

[151] Ren S, Yu Q, Yu X, et al. Graphene-supported metal single-atom catalysts: a concise review. Sci China Mater. 2020;63(6):903–920.

[152] Lei Y, Mehmood F, Lee S, et al. Increased silver activity for direct propylene epoxidation via subnanometer size effects. Science. 2010;328(5975):224–228. DOI:10.1126/science.1185200 20378815

[153] Yan H, Cheng H, Yi H, et al. Single-atom Pd^1^/graphene catalyst achieved by atomic layer deposition: remarkable performance in selective hydrogenation of 1, 3-butadiene. J Am Chem Soc. 2015;137(33):10484–10487. DOI:10.1021/jacs.5b06485 26268551

[154] Gao G, Jiao Y, Waclawik ER, et al. Single atom (Pd/Pt) supported on graphitic carbon nitride as an efficient photocatalyst for visible-light reduction of carbon dioxide. J Am Chem Soc. 2016;138(19):6292–6297. DOI:10.1021/jacs.6b02692 27116595

[155] Zhang H, Wei J, Dong J, et al. Efficient visible‐light‐driven carbon dioxide reduction by a single‐atom implanted metal–organic framework. Angew Chem. 2016;128(46):14522–14526. DOI:10.1002/ange.201608597 27736031

[156] Huang P, Huang J, Pantovich SA, et al. Selective CO_2_ reduction catalyzed by single cobalt sites on carbon nitride under visible-light irradiation. J Am Chem Soc. 2018;140(47):16042–16047. DOI:10.1021/jacs.8b10380 30415539

[157] Fu Q, Saltsburg H, Flytzani-Stephanopoulos M. Active nonmetallic Au and Pt species on ceria-based water-gas shift catalysts. Science. 2003;301(5635):935–938. 12843399 10.1126/science.1085721

[158] Qiao B, Wang A, Yang X, et al. Single-atom catalysis of CO oxidation using Pt-1/feo x. Nat Chem. 2011;3(8):634–641. DOI:10.1038/nchem.1095 21778984

[159] Chen W, Pei J, He CT, et al. Single tungsten atoms supported on MOF‐derived N‐doped carbon for robust electrochemical hydrogen evolution. Adv Mater. 2018;30(30):1800396. DOI:10.1002/adma.201800396 29888491

[160] Qiu HJ, Ito Y, Cong W, et al. Nanoporous graphene with single‐atom nickel dopants: an efficient and stable catalyst for electrochemical hydrogen production. Angewandte Chemie. 2015;54(47):14031–14035. DOI:10.1002/anie.201507381 26474177

[161] Ji S, Qu Y, Wang T, et al. Rare‐earth single erbium atoms for enhanced photocatalytic CO_2_ reduction. Angewandte Chemie. 2020;59(26):10651–10657. DOI:10.1002/anie.202003623 32189435

[162] Mane GP, Dhawale DS, Anand C, et al. Selective sensing performance of mesoporous carbon nitride with a highly ordered porous structure prepared from 3-amino-1,2,4-triazine [10.1039/C2TA01215D]. J Mater Chem A. 2013;1(8):2913–2920. DOI:10.1039/C2TA01215D

[163] Talapaneni SN, Singh G, Kim IY, et al. Nanostructured carbon nitrides for CO_2_ capture and conversion. Adv Mater. 2020;32(18):1904635. DOI:10.1002/adma.201904635 31608512

[164] Kim S, Singh G, Sathish C, et al. Tailoring the pore size, basicity, and binding energy of mesoporous C_3_N_5_ for CO_2_ capture and conversion. Chem – Asian J. 2021;16(23):3999–4005. DOI:10.1002/asia.202101069 34653318

[165] Park D-H, Lakhi KS, Ramadass K, et al. Energy efficient synthesis of ordered mesoporous carbon nitrides with a high nitrogen content and enhanced CO_2_ capture capacity. Chem Eur J. 2017;23(45):10753–10757. DOI:10.1002/chem.201702566 28677823

[166] Yang J-H, Kim S, Kim IY, et al. Highly enhanced photocatalytic hydrogen evolution activity of graphitic carbon nitride with 3D connected mesoporous structure. Sustainable Mater Technol. 2020;25:e00184.

[167] Lin L, Yu Z, Wang X. Crystalline carbon nitride semiconductors for photocatalytic water splitting. Angew Chem. 2019;131(19):6225–6236. 10.1002/anie.201809897 30345661

[168] Dias EM, Christoforidis KC, Francas L, et al. Tuning thermally treated graphitic carbon nitride for H_2_ evolution and CO_2_ photoreduction: the effects of material properties and mid-gap states. ACS Appl Energy Mater. 2018;1(11):6524–6534. DOI:10.1021/acsaem.8b01441

[169] Talapaneni SN, Mane GP, Mano A, et al. Synthesis of nitrogen‐rich mesoporous carbon nitride with tunable pores, band gaps and nitrogen content from a single aminoguanidine precursor. ChemSuschem. 2012;5(4):700–708. DOI:10.1002/cssc.201100626 22389323

[170] Ni D, Zhang Y, Shen Y, et al. Promoting condensation kinetics of polymeric carbon nitride for enhanced photocatalytic activities. Chin Chem Lett. 2020;31(1):115–118.

[171] Yue B, Li Q, Iwai H, et al. Hydrogen production using zinc-doped carbon nitride catalyst irradiated with visible light. Sci Technol Adv Mater. 2011;12(3):034401. DOI:10.1088/1468-6996/12/3/034401 27877392 PMC5090464

[172] Zhang L, Ding N, Hashimoto M, et al. Sodium-doped carbon nitride nanotubes for efficient visible light-driven hydrogen production. Nano Res. 2018;11(4):2295–2309. DOI:10.1007/s12274-017-1853-3

[173] Gao L-F, Wen T, Xu J-Y, et al. Iron-doped carbon nitride-type polymers as homogeneous organocatalysts for visible light-driven hydrogen evolution. ACS Appl Mater Interfaces. 2016;8(1):617–624. DOI:10.1021/acsami.5b09684 26650485

[174] Guo S, Deng Z, Li M, et al. Phosphorus‐doped carbon nitride tubes with a layered micro‐nanostructure for enhanced visible‐light photocatalytic hydrogen evolution. Angew Chem. 2016;128(5):1862–1866. DOI:10.1002/ange.201508505 26692105

[175] Liu Y, Zhang X, Chen Z, et al. Electrocatalytic reduction of nitrogen on FeAg/Si for ammonia synthesis: a simple strategy for continuous regulation of faradaic efficiency by controlling H+ ions transfer rate. Appl Catal B Environ. 2021;283:119606.

[176] Zhu Y, Marianov A, Xu H, et al. Bimetallic Ag–Cu supported on graphitic carbon nitride nanotubes for improved visible-light photocatalytic hydrogen production. ACS Appl Mater Interfaces. 2018;10(11):9468–9477. DOI:10.1021/acsami.8b00393 29465987

[177] Mane GP, Talapaneni SN, Lakhi KS, et al. Highly ordered nitrogen‐rich mesoporous carbon nitrides and their superior performance for sensing and photocatalytic hydrogen generation. Angewandte Chemie. 2017;56(29):8481–8485. DOI:10.1002/anie.201702386 28382643

[178] Talapaneni SN, Mane GP, Park D-H, et al. Diaminotetrazine based mesoporous C_3_N_6_ with a well-ordered 3D cubic structure and its excellent photocatalytic performance for hydrogen evolution. J Mater Chem A. 2017;5(34):18183–18192. DOI:10.1039/C7TA04041E

[179] Kim IY, Kim S, Jin X, et al. Ordered mesoporous C_3_N_5_ with a combined triazole and triazine framework and its graphene hybrids for the oxygen reduction reaction (ORR). Angew Chem. 2018;130(52):17381–17386. DOI:10.1002/ange.201811061 30407712

[180] Ma J, Peng X, Zhou Z, et al. Extended conjugation tuning carbon nitride for non-sacrificial H_2_O_2_ photosynthesis and hypoxic tumor therapy. Angew Chem Int Ed. 2022;61(43):e202210856. DOI:10.1002/anie.202210856 35939064

[181] Huang C, Wen Y, Ma J, et al. Unraveling fundamental active units in carbon nitride for photocatalytic oxidation reactions. Nat Commun. 2021;12(1):12. 33436603 10.1038/s41467-020-20521-5 PMC7804405

[182] Wang J, Zhou Q, Shen Y, et al. Carbon nitride Co-catalyst activation using N-doped carbon with enhanced photocatalytic H_2_ evolution. Langmuir. 2019;35(38):12366–12373. 31464446 10.1021/acs.langmuir.9b01796

[183] Dong G, Zhao K, Zhang L. Carbon self-doping induced high electronic conductivity and photoreactivity of g-C_3_N_4_. Chem Comm. 2012;48(49):6178–6180. 22588283 10.1039/c2cc32181e

[184] Zhao Z, Sun Y, Dong F, et al. Template synthesis of carbon self-doped g-C_3_N_4_ with enhanced visible to near-infrared absorption and photocatalytic performance. RSC Adv. 2015;5(49):39549–39556. DOI:10.1039/C5RA03433G

[185] Fang J, Fan H, Li M, et al. Nitrogen self-doped graphitic carbon nitride as efficient visible light photocatalyst for hydrogen evolution. J Mater Chem A. 2015;3(26):13819–13826. DOI:10.1039/C5TA02257F

